# Supplementary material for: A universal strategy for AAV delivery of base editors to correct genetic point mutations in neonatal PKU mice
Source: Mol Ther Methods Clin Dev. 2022 Jan 7;24:230–40. doi: 10.1016/j.omtm.2022.01.001 (PMC8803597; doi:10.1016/j.omtm.2022.01.001)
Supplement: Document S1. Figures S1–S6, Tables S1–S5, and Note S1 [file mmc1.pdf]

OMTM, Volume *24*

Supplemental information

A universal strategy for AAV delivery of base editors to correct genetic point mutations in neonatal PKU mice

Lifang Zhou, Jing Su, Jie Long, Rui Tao, Wenling Tang, Fengming Qin, Nan Liu, Yanhong Wang, Yaoge Jiao, Yun Hu, Lurong Jiang, Li Li, Yang Yang, and Shaohua Yao

Supplementary information

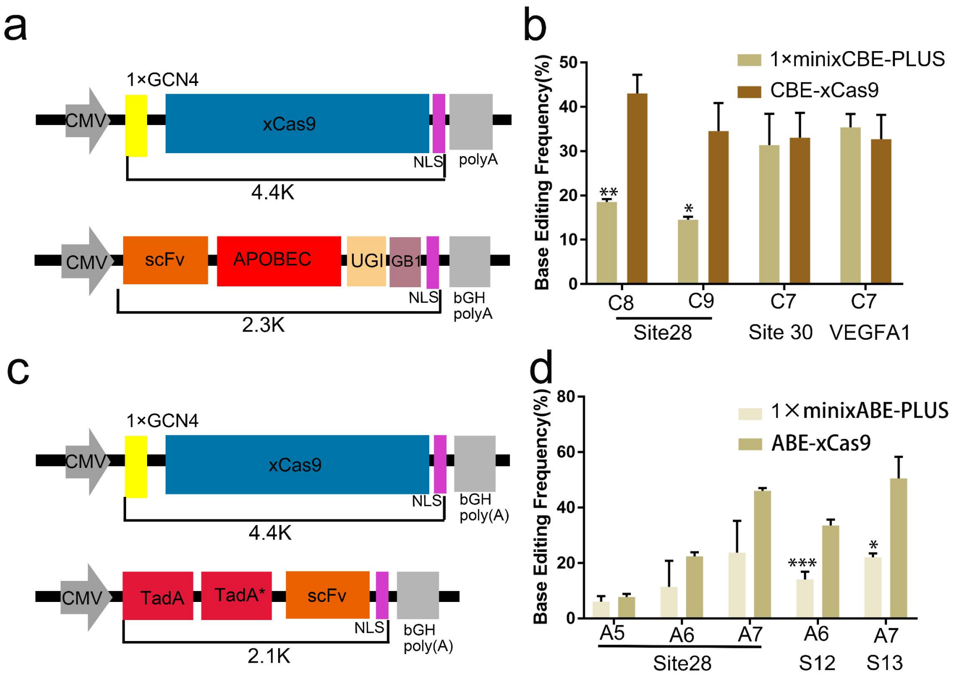

Figure S1. Construction and verification of minixBE-PLUS editing system. (a) Schematic diagram showing the expression cassettes of pCMV-1×GCN4-xCas9, CMV-scFv-CBE. (b) Cytosine base editing of 1×minixCBE-PLUS and CBE-xCas9 at three genomic loci in HEK293T cells. (n = 3 independent experiments). Values represent mean ± SD. Asterisks indicate statistically significant differences in editing efficiencies observed between CBE-xCas9 and 1×minixCBE-PLUS at each site. (c) Schematic diagram showing the expression cassettes of pCMV-1×GCN4-xCas9, pCMV-ABE-scFv. (d) Adenine base editing of 1×minixABE-PLUS and ABE-xCas9 at three genomic loci in HEK293T cells. (n = 3 independent experiments). Values represent mean ± SD. Asterisks indicate statistically significant differences in editing efficiencies observed between ABE-xCas9 and 1×minixABE-PLUS at each site. (abbreviations, CMV(human cytomegalovirus promoter), UGI(Uracil DNA

glycosylase inhibitor), NLS (nuclear localization signal), GB1(G protein B1 domain))

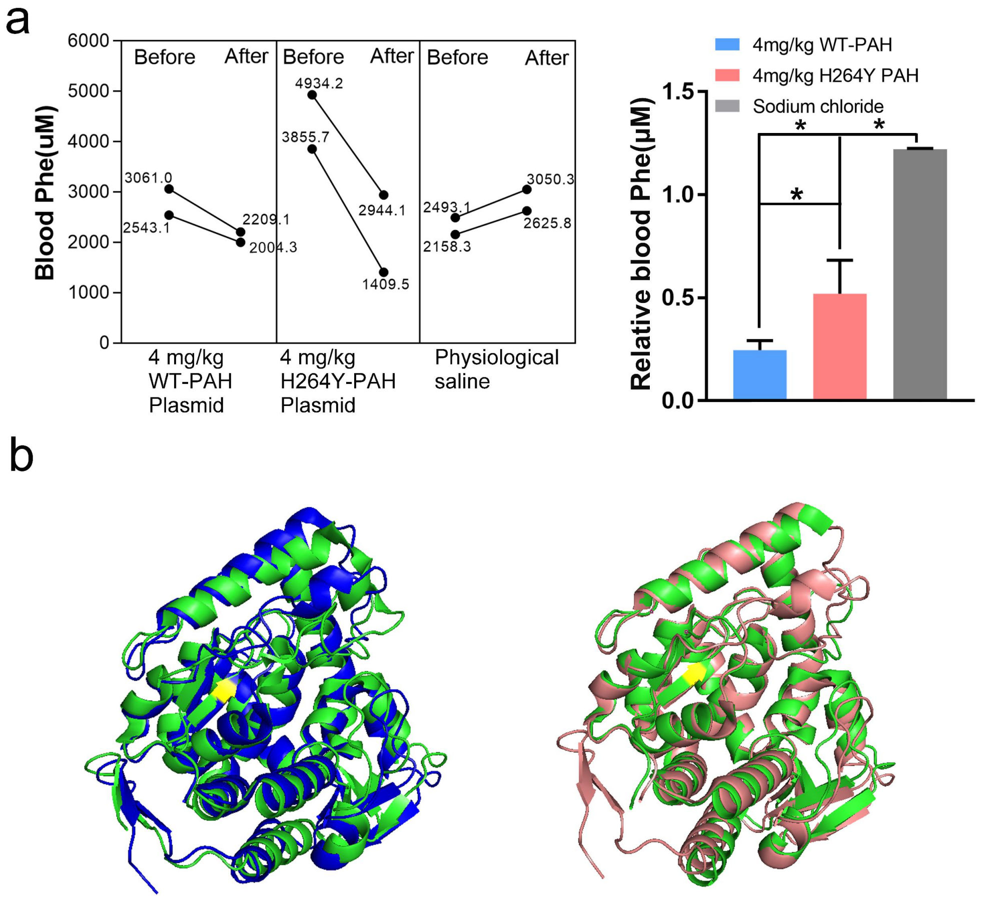

Figure S2. PAH activity evaluation. (a) H264Y-Pah enzyme activity verification. Changes of blood Phe in mice after plasmid injection (left panel). The relative changes of Phe in mice blood after plasmid injection (right panel) (n = 2 or 3 independent experiments). Values represent mean ± SD. Asterisks indicate statistically significant differences in functional editing efficiencies observed between WT-PAH or H254Y-PAH plasmid injected mice and sodium chloride injected mice. (b) Comparison of protein structure between phenylalanine hydroxylase monomer (PDB, 6hpo) (green) and tyrosine hydroxylase monomer (blue) (PDB, 3hfb), or

tryptophan hydroxylase monomer(pink) (PDB, 13e2t). position F264 of phenylalanine hydroxylase is marked in yellow.

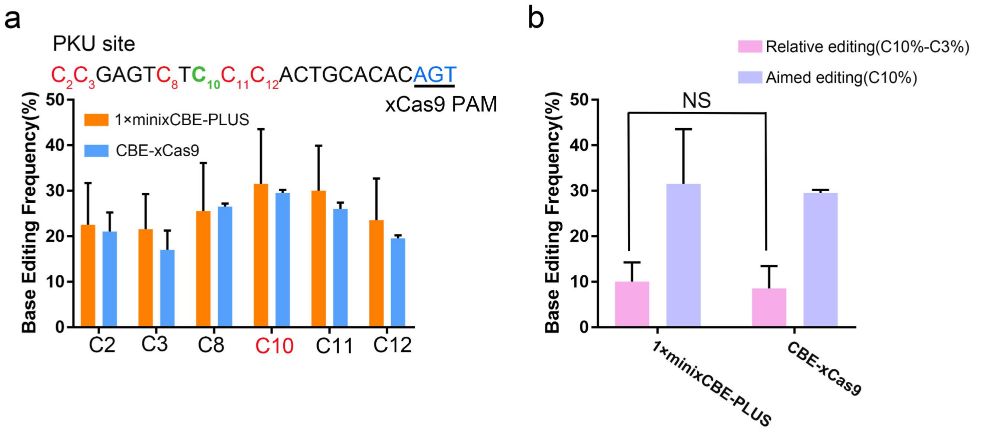

Figure S3. *In vitro* base editing of mouse PKU site by 1×minixCBE-PLUS. (a) The editing of 1×minixCBE-PLUS, and CBE-xCas9 with the sgRNA on PKU site of each c (n = 3 independent experiments). The aimed cytosine (C10, i.e. C835) was shown in green, and the bystanders were shown in red. (b) The relative editing efficiency (C10%-C3%) of 1×minixCBE-PLUS, and CBE-xCas9 (n = 3 independent experiments). Values represent mean ± SD. Asterisks indicate statistically significant differences in functional editing efficiencies observed between CBE-xCas9 and 1×minixCBE-PLUS at PKU site .

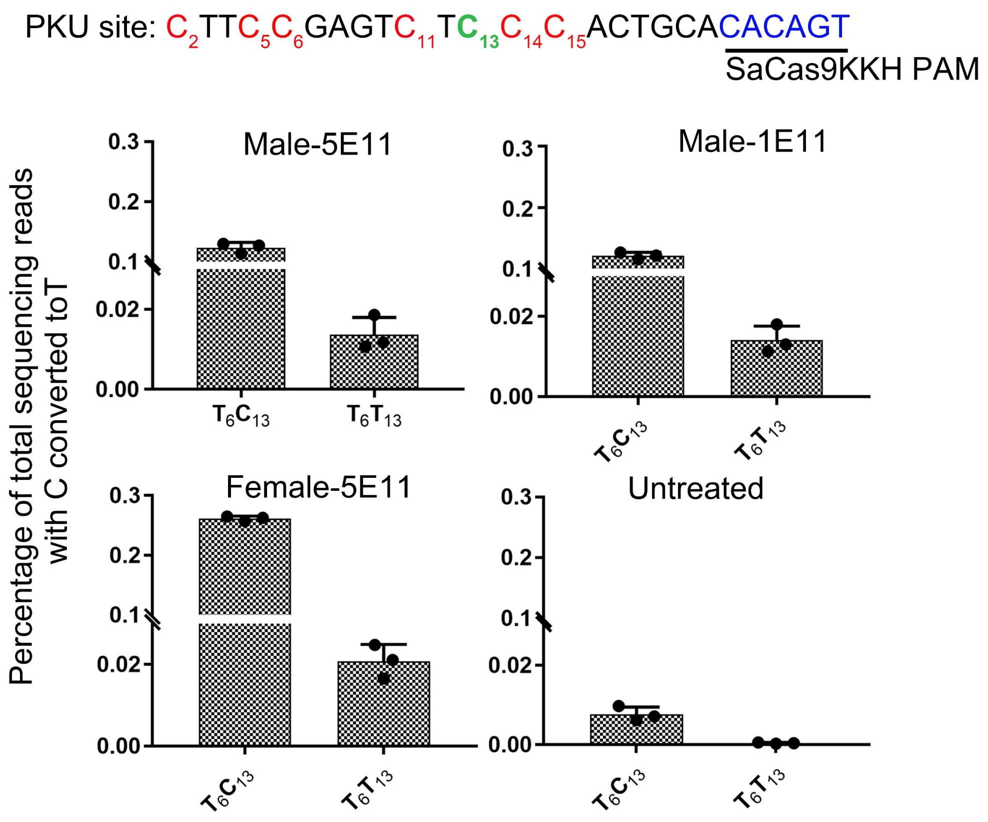

Figure S4. AAV8-miniSaCBE-PLUS-PKU produced minus non-sense edits *in vivo.* HTS of high-dose (5E11-PKU) male, low-dose (1E11-PKU) male, high-dose (5E11-PKU) female, untreated mice liver DNA revealed that AAV8-miniSaCBE-PLUS-PKU only produced minus non-sense edits *in vivo.*

	Phe										Weigh									
	Male					Female					Male					Female				
Groups / Weeks	8w	12w	16w	20w	24w	8w	12w	16w	20w	24w	8w	12w	16w	20w	24w	8w	12w	16w	20w	24w
WT or PKU heterozygote vs 5E11 AAV8-miniSaCBE-PLUS-PKU	ns	ns	ns	ns	ns	ns	ns	ns	ns	ns	ns	ns	ns	ns	ns	**	*	ns	ns	ns
WT or PKU heterozygote vs 1E11 AAV8-miniSaCBE-PLUS-PKU	****	*	ns	ns	ns						**	ns	ns	ns	ns					
WT or PKU heterozygote vs 5E11 AAV8-miniSaCBE-PLUS-ctrl	****	****	****	****	****	****	****	****	****	****	****	****	**	***	****	****	****	***	**	***
WT or PKU heterozygote vs PKU untreated	****	****	****	****	****	****	****	****	****	****	***	**	ns	*	**	****	****	****	****	****
5E11 AAV8-miniSaCBE-PLUS-PKU vs 1E11 AAV8-miniSaCBE-PLUS-PKU	****	*	ns	ns	ns						*	ns	ns	ns	ns					
5E11 AAV8-miniSaCBE-PLUS-PKU vs 5E11 AAV8-miniSaCBE-PLUS-ctrl	****	****	****	****	****	****	****	****	****	****	****	**	ns	ns	ns	**	***	***	***	***
5E11 AAV8-miniSaCBE-PLUS-PKU vs PKU untreated	****	****	****	****	****	****	****	****	****	****	**	ns	ns	ns	ns	ns	**	****	****	****
1E11 AAV8-miniSaCBE-PLUS-PKU vs 5E11 AAV8-miniSaCBE-PLUS-ctrl	ns	ns	**	****	****						ns	ns	*	*	*					
1E11 AAV8-miniSaCBE-PLUS-PKU vs PKU untreated	ns	****	***	****	****						ns	ns	ns	ns	ns					
5E11 AAV8-miniSaCBE-PLUS-ctrl vs PKU untreated	ns	****	ns	ns	ns	ns	ns	*	ns	ns	ns	ns	ns	ns	ns	ns	ns	ns	ns	ns

Figure S5. The results of *ANOVA* analysis between the mutiple groups in

Figure 4e and Figure 4f. Asterisks indicate statistically significant differences in phe level and weigh of tested mice between multiple groups.

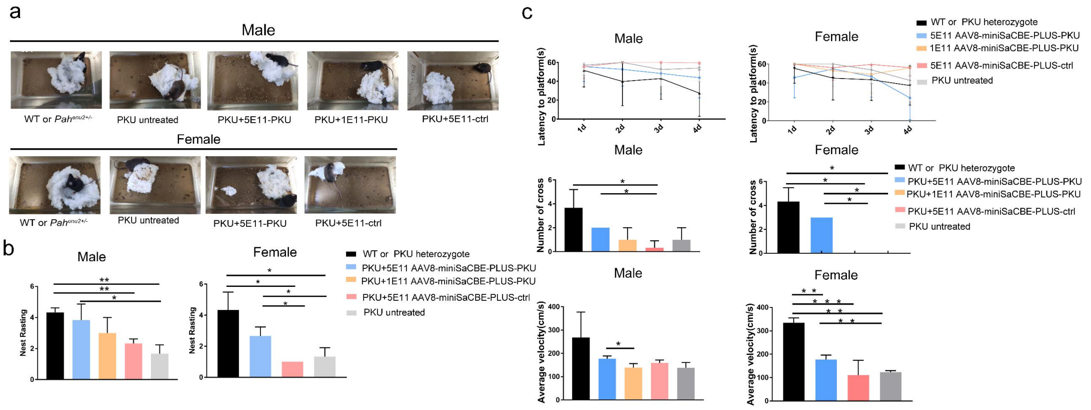

Figure S6. Behavioral analysis. (a) Photos of male and female mice' nesting situation. (b) Rating assessment of mice's nesting ability. n=3 mice for each group. Values represent mean ± SD. Asterisks indicate statistically significant differences between each group. (c) Performance in the water maze is presented with male and female respectively. The results are shown in top latency to the target platform (seconds), middle number of crosses, bottom average velocity (cm/s). n=3 mice for each group. Values represent mean ± SD. Asterisks indicate statistically significant differences between each group.

Table S1. List of sgRNAs and Oligos sequence

sgRNA	target sequence	Oligo F	Oligo R
FANCF site1	GATGTTCCAATCAGTACGCA **GAGAGT**	CACCGATGTTCCAATCAGTACGCA	AAACTGCGTACTGATTGGAACATC
CYP4V2 site4	TGCCTTAGATATCATCTGTG **GAGAAT**	CACCGTGCCTTAGATATCATCTGTG	AAACCACAGATGATATCTAAGGCAC
HEK3-1	TCTGCTTCTCCAGCCCTGGC **CTGGGT**	CACCGTCTGCTTCTCCAGCCCTGGC	AAACGCCAGGGCTGGAGAAGCAGAC
HEK293 site4-4	CTTTAACCCCCACCTCCAGC **CGCAGT**	CACCGCTTTAACCCCCACCTCCAGC	AAACGCTGGAGGTGGGGGTTAAAGC
EMX1	CCTCCCTCCCTGGCCCAGGT **GAAGGT**	CACCGCCTCCCTCCCTGGCCCAGGT	AAACACCTGGGCCAGGGAGGGAGGC
FANCF site2	GCCGTCTCCAAGGTGAAAGC **GGAAGT**	CACCGCCGTCTCCAAGGTGAAAGC	AAACGCTTTCACCTTGGAGACGGC
Sa site6	GATGTTCCAATCAGTACGCA **GAGAGT**	CACCGATGTTCCAATCAGTACGCA	AAACTGCGTACTGATTGGAACATCC
VEGFA site11	GCTCCATTCACCCAGCTTCCC **TGTGGT**	CACCGCTCCATTCACCCAGCTTCCC	AAACGGGAAGCTGGGTGAATGGAGC
RUNX1 4	GTACTCACCTCTCATGAAGCACT **GTGGGT**	CACCGTACTCACCTCTCATGAAGCACT	AAACAGTGCTTCATGAGAGGTGAGTAC
EMX1 #6	GCAACCACAAACCCACGAGGG **CAGAGT**	CACCGCAACCACAAACCCACGAGGG	AAACCCCTCGTGGGTTTGTGGTTGC
Sa site1	CTGAATAGCTGCAAACAAGTG **CAGAAT**	CACCGCTGAATAGCTGCAAACAAGTG	AAACCACTTGTTTGCAGCTATTCAGC
Sa site11	GCTGTTGCATGAGGAAAGGGAC **TAGAGT**	CACCGCTGTTGCATGAGGAAAGGGAC	AAACGTCCCTTTCCTCATGCAACAGC
Site28	GACAAACCAGAAGCCGCTCC **TGG**	CACCGACAAACCAGAAGCCGCTCC	AAACGGAGCGGCTTCTGGTTTGTCC
Site30	GAACACAAAGCATAGACTGC **GGG**	CACCGAACACAAAGCATAGACTGC	AAACGCAGTCTATGCTTTGTGTTCC

VEGFA 1	GATGTCTGCAG GCCAGATGA **GGG**	CACCGATGTCTG CAGGCCAGATGA	AAACTCATCTGGCCT GCAGACATC
S12	GCAGACGGCAG TCACTAGGG **GGC**	CACCGCAGACGG CAGTCACTAGGG	AAACCCCTAGTGACT GCCGTCTGC
S13	GTCGCAGGACA GCTTTTCCT **AGA**	CACCGTCGCAGG ACAGCTTTTCCT	AAACAGGAAAAGCTG TCCTGCGAC
18-PKU-sgRNA	TCCGAGTCTCC CACTGCA **CACAGT**	CACCGTCCGAGT CTTCCACTGCA	AAACTGCAGGGGAAG ACTGGAC
19-PKU-sgRNA	TTCCGAGTCTCC CACTGCA **CACAGT**	CACCGTTCCGAG TCTTCCACTGCA	AAACTGCAGGGGAAG ACTGGAAC
20-PKU-sgRNA	CTTCCGAGTCTC CCACTGCA **CACAGT**	CACCGCTTCCGA GTCTTCCACTGC A	AAACTGCAGGGGAAG ACTGGAAGC
21-PKU-sgRNA	CCTTCCGAGTCT TCCACTGCA **CACAGT**	CACCGCCTTCCG AGTCTTCCACTG CA	AAACTGCAGTGGAAG ACTGGAAGGC

Table S2. Primers used to amplify each target regions for sanger sequencing

Target site	chrom	Forward primer	Reverse primer
FANCF site1	Chr11	CGACGAGACAAAGGCG GCT	TCACTGTGACGTCCTGCT CT
CYP4V2 site4	Chr4	TGAAGAAACTTCGGTAT CTG	TATGGAAGCCCATGCAG GCG
HEK3-1	Chr9	AGAATGGGTCACAGTG GCAA	TAGGAAAAGCTGTCCTG CGA
HEK293 site4-4	Chr20	CTGCTGAGGGCGGCTTC TC	TGAAATCGCTCGGAGCC TC
EMX1	Chr2	TCTCTCTGGCCCACTGT GTC	CCATTGGCCTGCTTCGTG
FANCF site2	Chr11	CGACGAGACAAAGGCG GCT	TCACTGTGACGTCCTGCT CT
Sa site6	Chr11	ATGACTGGCATCATCTC GCA	GGTGCTGACGTAGGTAG TGC
VEGFA11	Chr6	GGAACAAGGGCCTCTGT CTG	GCCGTTCCCTCTTTGCTA GG
RUNX14	Chr2	GTTCTCACGCACCGACT	GAGTCCCAGAGGTATCC

	1	GAA	AGC
EMX1 #6	Chr2	TCTCTCTGGCCCACTGTGTC	CCATTGGCCTGCTTCGTG
Sa site1	Chr5	AAGTTACTGCAGCCCAAG	CAAGCAGGTGATTACAGG
Sa site11	Chr1	GCAGAAACCACAGTGTGT	CACTACCCCTGTTCCTAAAG
Site28	Chr3	GGCACAAAGGATGAAGGCT	GCTCAGTCTTGCATGAAACAC
Site30	Chr5	ACAGGCTACCCCCTAAGT	TCCCAAGTGAGAAGCCAGTG
VEGFA site1	Chr6	GGAACAAGGGCCTCTGTCTG	GCCGTTCCCTCTTTGCTAGG
S12	Chr6	CACAGCTTCCCGTTCTCAGC	AGGGACACACAGATCTATT
S13	Chr9	ATGTGGGCTGCCTAGAAAGG	CCCAGCCAAACTTGTCAACC
PKU-sgRNA		CCTTGGGGAGTCATACCTCA	ATAAAGCAGGCAGTGGATCA
BGH_PA_qPCR		GCCAGCCATCTGTTGT	GGAGTGGCACCTTCCA
BGH_PA_qPCR_Probe		TCCCCCGTGCCTTCCTTGACC	

Table S3. HTS Primers used for on-target sequence amplification.

Primer name	Forward primer	Reverse primer
1-Bar-DNA-OT	aagcgtg CCTTGGGGAGTCATACCTCA	GTTCAGGTGTGTACATGG
2-Bar-DNA-OT	attctcg CCTTGGGGAGTCATACCTCA	
3-Bar-DNA-OT	attgctc CCTTGGGGAGTCATACCTCA	
4-Bar-DNA-OT	atcaagc CCTTGGGGAGTCATACCTCA	
5-Bar-DNA-OT	atccgga CCTTGGGGAGTCATACCTCA	
6-Bar-DNA-OT	atcgaag CCTTGGGGAGTCATACCTCAA	
7-Bar-DNA-OT	atcgtcc CCTTGGGGAGTCATACCTCA	
8-Bar-DNA-OT	atgcttc CCTTGGGGAGTCATACCTCA	
9-Bar-DNA-OT	atggcga CCTTGGGGAGTCATACCTCA	
10-Bar-DNA-OT	acttagc CCTTGGGGAGTCATACCTCA	
S99-Bar-DNA-OT	tgtgtca CCTTGGGGAGTCATACCTCA	
S38-Bar-DNA-OT	caactgt CCTTGGGGAGTCATACCTCA	
1-RNA-pku-ontargetF	catcgac GACAACATCCCGCAGCT	GTTCAGGTGTGTACATGG
2-RNA-pku-ontargetF	catgatc GACAACATCCCGCAGCT	
3-RNA-pku-ontargetF	catgcaa GACAACATCCCGCAGCT	
4-RNA-pku-ontargetF	cagttac GACAACATCCCGCAGCT	

5-RNA-pku-ontargetF	ctatggt GACAACATCCCGCAGCT
6-RNA-pku-ontargetF	ctacaag GACAACATCCCGCAGCT
7-RNA-pku-ontargetF	ctacgtc GACAACATCCCGCAGCT
8-RNA-pku-ontargetF	ctagcac GACAACATCCCGCAGCT
9-RNA-pku-ontargetF	cttagct GACAACATCCCGCAGCT
10-RNA-pku-ontargetF	cttggag GACAACATCCCGCAGCT
11-RNA-pku-ontargetF	ctgctaa GACAACATCCCGCAGCT
12-RNA-pku-ontargetF	ctcgtat GACAACATCCCGCAGCT
13-RNA-pku-ontargetF	gaactac GACAACATCCGCAGCT
14-RNA-pku-ontargetF	gaaccta GACAACATCCCGCAGCT
15-RNA-pku-ontargetF	gaaggtc GACAACATCCCGCAGCT
16-RNA-pku-ontargetF	gattctg GACAACATCCCGCAGCT
17-RNA-pku-ontargetF	gattgct GACAACATCCCGCAGCT
18-RNA-pku-ontargetF	gatgacg GACAACATCCCGCAGCT
19-RNA-pku-ontargetF	gatgtgc GACAACATCCCGCAGCT
20-RNA-pku-ontargetF	gaccatc GACAACATCCCGCAGCT
21-RNA-pku-ontargetF	gacgaat GACAACATCCCGCAGCT

Table S4. Off-target sequence

Target site	sequence	chrom	strand
OT1	ACTTCAGACTCTCTCACTGCAACTGGT	Chr14	-
OT2	ATTTCCAAGTCTCCCACTGCTGTGAGT	Chr10	+
OT3	CCTCCCCAGTCTCCCACTGCTGACAAT	Chr11	-
OT4	CCTACAGAGTATTCCACTGCACCCAGT	Chr10	-
OT5	CCTTTCGAGTCTTTCACTGCACTCAGT	Chr7	-
OT6	TCTTCCTAGGCTCCCACAGCAATGAGT	Chr11	-
OT7	CATTCCCAGTGTACCACTGCAGTCAGT	Chr4	+
OT8	TCTTCAGAGTCTTCCTCTGCATCCGAT	ChrX	+

Table S5. Off-target Primers for amplification

off-target site	Forward primer	Reverse primer
OT1	gctgtaa GCATTTCTCAGATCTAATCTTCTGA	TGAGCATATGTGACCT GAGGA
OT2	gcctcta GAAACAGTTTGCACCCCCTA	CCCCAGGTTCATCAGT TCAC
OT3	gcgagtt CTTAGCGCCGCTGACTCT	TCCCTCTCCTTTTGCTT CCT
OT4	aactagg GACTGCAATACTCACGGTTCC	TGAGCCCAAGAGACTT CCTG
OT5	aacctag GCCATTTGATGAATGAATAGCA	GGTTTTCCATCCGTCC TGT

OT6	aagatgc AGGATGAGACCCAGGACCA	CCTGCAAGCTTTCCAC TCAT
OT7	ctggatg GCTGGGGCTATACAAAATTCC	CAGTAGTTCCAGTCAC GGTTG
OT8	ccaatga AAAAATATGCCCGTGTCATTG	TGCTGCTGTTGGAATC TGAG

Note1:

Plasmid sequences (CDS)

4×GCN4-SaCas9KKH

ATGGAGGAACTGCTGAGCAAGAACTACCACCTGGAAAACGAGGTGGCCA
GACTGAAGAAAGGCTCTGGCTCTGGCGGAAGCGGTTCTGGCGGATCAGGA
TCTGGAAGTGGCGGCTCTGGAAGCGGAGGTTCTGGATCTGGCGAAGAACT
GCTGTCTAAGAATTATCACCTCGAGAACGAAGTGGCTCGGCTCAAGAAAG
GCAGTGGCAGCGGAGGAAGTGGATCCGGCGGTAGTGGTAGTGGAAGCGG
CGGATCCGGCTCAGGCGGATCTGGTTCAGGGGAAGAACTCCTCTCCAAAA
ACTACCATCTCGAGAATGAGGTCGCCCGCCTGAAAAAAGGATCAGGTTCC
GGTGGTTCTGGCAGCGGTGGTTCAGGCTCAGGCAGCGGCGGTAGCGGTAG
CGGTGGAAGCGGAAGCGGAGAAGAACTTCTCAGCAAAAATTACCACTTG
GAGAATGAAGTTGCAAGACTCAAAAAAGGTTCCGGCAGTGGCGGCAGCG
GCAGCGGCGGATCTGGTAGTGGATCTGGTGGCAGTGGTTCAGGCGGAAGT
GGTAGCGGACCCGGGACCTCAGAGTCCGCCACACCCGAAAGTGGGAAAC
GGAACTACATCCTGGGGCTTGCCATTGGGATAACCAGCGTTGGCTACGGA
ATTATTGATTATGAGACACGCGATGTGATTGACGCCGGGGTTAGGCTGTTC
AAAGAGGCCAACGTTGAAAACAACGAGGGAAGACGGAGTAAGCGCGGA
GCAAGAAGACTCAAGCGCAGACGGAGACATCGGATTCAGAGGGTGAAAA
AGCTGCTCTTCGATTACAATCTCCTGACCGATCATAGTGAGCTGAGCGGA
ATCAACCCCTACGAGGCGCGAGTGAAAGGGCTTTCCCAGAAGCTGTCCGA
AGAGGAGTTCTCCGCCGCGTTGCTGCACCTGGCCAAACGGAGGGGGGTTC
ACAATGTAAACGAAGTGGAGGAGGACACGGGCAATGAACTTAGTACGAA
AGAACAGATCAGTAGGAACTCTAAGGCTCTCGAAGAGAAATACGTCGCTG
AGTTGCAGCTTGAGAGACTGAAAAAAGACGGCGAAGTACGCGGATCTATT
AATAGGTTCAAGACTTCAGATTACGTAAAGGAAGCCAAGCAGCTCCTGAA
AGTACAGAAAGCGTACCATCAGCTCGATCAGAGCTTCATCGATACCTACA
TAGATTTGCTGGAGACACGGAGGACATACTACGAGGGCCCAGGGGAAGG
ATCTCCTTTTGGGTGGAAGGACATCAAGGAATGGTACGAGATGCTTATGG
GACATTGTACATATTTTCCGGAGGAGCTCAGGAGCGTCAAGTACGCCTAC
AATGCCGACCTGTACAATGCCCTCAATGACCTCAATAACCTCGTGATTACC
AGGGACGAGAACGAGAAGCTGGAGTACTATGAAAAGTTCCAGATTATCG
AGAATGTGTTTAAGCAGAAGAAGAAGCCGACACTTAAGCAGATTGCAAA
GGAAATCCTCGTGAATGAGGAAGATATCAAGGGATACAGAGTGACAAGT
ACAGGCAAGCCCGAGTTCACAAATCTGAAGGTGTACCACGATATTAAGGA
CATAACCGCACGAAAGGAGATAATCGAAAACGCTGAGCTCCTCGATCAG

ATCGCAAAAATTCTTACCATCTACCAGTCTAGTGAGGACATTCAGGAGGA
ACTGACTAATCTGAACAGTGAGCTCACCCAAGAGGAAATTGAGCAGATTT
CAAACCTGAAAGGCTACACCGGGACGCACAATCTGAGCCTCAAAGCAATC
AACCTCATTCTGGATGAACTTTGGCACACAAATGACAACCAAATTGCCAT
ATTCAACCGCCTGAAACTGGTGCCAAAAAAAGTGGATCTGTCACAGCAAA
AGGAAATCCCTACAACCTTGGTTGACGATTTTATTCTGTCCCCCGTTGTCA
AGCGGAGCTTCATCCAGTCAATCAAGGTGATCAATGCCATCATTAAAAAA
TACGGATTGCCAAACGATATAATTATCGAGCTTGCACGAGAGAAGAACTC
AAAGGACGCCCAGAAGATGATTAACGAAATGCAGAAGCGCAACCGCCAG
ACAAACGAACGCATAGAGGAAATTATAAGAACAACCGGCAAAGAGAATG
CCAAGTATCTGATCGAGAAAATCAAGCTGCACGACATGCAAGAAGGCAA
GTGCCTGTACTCTCTGGAAGCTATCCCACTCGAAGATCTGCTGAATAATCC
ATTCAATTACGAGGTGGACCACATCATCCCTAGATCCGTAAGCTTTGACA
ATTCCTTCAATAACAAAGTTCTGGTTAAACAGGAGGAAAATTCTAAAAAA
GGGAACCGGACCCCGTTCCAGTACCTGAGCTCCAGTGACAGCAAGATTAG
CTACGAGACTTTTAAGAAACATATTCTGAATCTGGCCAAAGGCAAAGGCA
GGATCAGCAAGACCAAGAAGGAGTACCTCCTCGAAGAACGCGACATTAA
CAGATTTAGTGTGCAGAAAGATTTCATCAACCGAAACCTTGTCGATACTC
GGTACGCCACGAGAGGCCTGATGAATCTCCTCAGGAGCTACTTCCGCGTC
AATAATCTGGACGTTAAAGTCAAGAGCATAAATGGGGGATTCACCAGCTT
TCTGAGGAGAAAGTGGAAGTTTAAGAAGGAACGAAACAAAGGATACAAG
CACCATGCTGAGGATGCTTTGATCATCGCTAACGCGGACTTTATCTTTAAG
GAATGGAAAAAGCTGGATAAGGCAAAGAAAGTGATGGAAAACCAGATGT
TCGAGGAGAAGCAGGCAGAGTCAATGCCTGAGATCGAGACAGAGCAGGA
ATACAAGGAAATTTTCATCACCCCTCATCAGATTAAACACATAAAGGACT
TCAAAGACTATAAATACTCTCATAGGGTGGACAAAAAACCCAATCGCAAG
CTCATTAATGACACCCTGTACTCAACACGGAAGGATGATAAAGGTAATAC
CTTGATTGTGAATAATCTTAATGGATTGTATGACAAAGATAACGACAAGC
TCAAGAAGCTGATCAACAAGTCTCCAGAGAAGCTCCTTATGTATCACCAC
GACCCACAGACTTATCAGAAATTGAAACTGATCATGGAGCAATACGGGGA
TGAGAAGAACCCACTCTACAAATATTATGAGGAAACAGGTAATTACCTGA
CCAAGTACTCCAAGAAGGATAACGGACCAGTGATCAAAAAGATAAAGTA
CTATGGCAACAAACTTAATGCGCATTTGGACATAACTGACGATTACCCCA
ATTCTCGAAACAAGGTTGTGAAGCTCTCCCTGAAGCCTTATAGATTTGACG
TGTACCTGGATAATGGGGTTTATAAATTCGTCACCGTGAAAAATCTGGAC
GTGATCAAAAAGGAGAACTATTATGAAGTAAACTCAAAGTGCTATGAGGA
GGCGAAGAAGCTGAAGAAGATCTCCAATCAGGCCGAGTTCATCGCTTCCT
TCTATAAGAACGATCTCATCAAGATCAATGGAGAGCTTTATCGCGTCATT
GGTGTGAACAATGACTTGCTGAACAGGATCGAAGTCAATATGATAGACAT
TACCTACCGGGAGTATCTCGAAAACATGAATGATAAACGGCCGCCTCACA
TCATCAAGACAATCGCATCTAAAACTCAGTCAATAAAAAAGTACTCTACC
GATATCCTGGGGAATCTCTATGAAGTGAAGTCAAAGAAGCACCCACAAAT
CATTAAAAAAGGTGGATCCCCCAAGAAGAAGAGGAAAGTCTCGAGCGAC
TACAAAGACCATGACGGTGATTATAAAGATCATGACATCGATTACAAGGA

TGACGATGACAAGTCTGGTGGTTCTACTAATCTGTCAGATATTATTGAAAA
GGAGACCGGTAAGCAACTGGTTATCCAGGAATCCATCCTCATGCTCCCAG
AGGAGGTGGAAGAAGTCATTGGGAACAAGCCGGAAAGCGATATACTCGT
GCACACCGCCTACGACGAGAGCACCGACGAGAATGTCATGCTTCTGACTA
GCGACGCCCCTGAATACAAGCCTTGGGCTCTGGTCATACAGGATAGCAAC
GGTGAGAACAAGATTAAGATGCTCTCTGGTGGTTCTCCCAAGAAGAAGAG
GAAAGTCTAA

2×GCN4-SaCas9KKH

ATGGAGGAACTGCTGAGCAAGAACTACCACCTGGAAAACGAGGTGGCCA
GACTGAAGAAAGGCTCTGGCTCTGGCGGAAGCGGTTCTGGCGGATCAGGA
TCTGGAAGTGGCGGCTCTGGAAGCGGAGGTTCTGGATCTGGCGAAGAACT
GCTGTCTAAGAATTATCACCTCGAGAACGAAGTGGCTCGGCTCAAGAAAG
GCAGTGGCAGCGGAGGAAGTGGATCCGGCGGTAGTGGTAGTGGAAGCGG
CGGATCCGGCTCAGGCGGATCTGGTTCAGGGCCCGGGACCTCAGAGTCCG
CCACACCCGAAAGTGGGAAACGGAACTACATCCTGGGGCTTGCCATTGGG
ATAACCAGCGTTGGCTACGGAATTATTGATTATGAGACACGCGATGTGAT
TGACGCCGGGGTTAGGCTGTTCAAAGAGGCCAACGTTGAAAACAACGAG
GGAAGACGGAGTAAGCGCGGAGCAAGAAGACTCAAGCGCAGACGGAGA
CATCGGATTCAGAGGGTGAAAAAGCTGCTCTTCGATTACAATCTCCTGAC
CGATCATAGTGAGCTGAGCGGAATCAACCCCTACGAGGCGCGAGTGAAA
GGGCTTTCCCAGAAGCTGTCCGAAGAGGAGTTCTCCGCCGCGTTGCTGCA
CCTGGCCAAACGGAGGGGGGTTCACAATGTAAACGAAGTGGAGGAGGAC
ACGGGCAATGAACTTAGTACGAAAGAACAGATCAGTAGGAACTCTAAGG
CTCTCGAAGAGAAATACGTCGCTGAGTTGCAGCTTGAGAGACTGAAAAAA
GACGGCGAAGTACGCGGATCTATTAATAGGTTCAAGACTTCAGATTACGT
AAAGGAAGCCAAGCAGCTCCTGAAAGTACAGAAAGCGTACCATCAGCTC
GATCAGAGCTTCATCGATACCTACATAGATTTGCTGGAGACACGGAGGAC
ATACTACGAGGGCCCAGGGGAAGGATCTCCTTTTGGGTGGAAGGACATCA
AGGAATGGTACGAGATGCTTATGGGACATTGTACATATTTTCCGGAGGAG
CTCAGGAGCGTCAAGTACGCCTACAATGCCGACCTGTACAATGCCCTCAA
TGACCTCAATAACCTCGTGATTACCAGGGACGAGAACGAGAAGCTGGAGT
ACTATGAAAAGTTCCAGATTATCGAGAATGTGTTTAAGCAGAAGAAGAAG
CCGACACTTAAGCAGATTGCAAAGGAAATCCTCGTGAATGAGGAAGATAT
CAAGGGATACAGAGTGACAAGTACAGGCAAGCCCGAGTTCACAAATCTG
AAGGTGTACCACGATATTAAGGACATAACCGCACGAAAGGAGATAATCG
AAAACGCTGAGCTCCTCGATCAGATCGCAAAAATTCTTACCATCTACCAG
TCTAGTGAGGACATTCAGGAGGAACTGACTAATCTGAACAGTGAGCTCAC
CCAAGAGGAAATTGAGCAGATTTCAAACCTGAAAGGCTACACCGGGACG
CACAATCTGAGCCTCAAAGCAATCAACCTCATTCTGGATGAACTTTGGCA
CACAAATGACAACCAAATTGCCATATTCAACCGCCTGAAACTGGTGCCAA
AAAAAGTGGATCTGTCACAGCAAAAGGAAATCCCTACAACCTTGGTTGAC
GATTTTATTCTGTCCCCCGTTGTCAAGCGGAGCTTCATCCAGTCAATCAAG
GTGATCAATGCCATCATTAAAAAATACGGATTGCCAAACGATATAATTAT

CGAGCTTGCACGAGAGAAGAACTCAAAGGACGCCCAGAAGATGATTAAC
GAAATGCAGAAGCGCAACCGCCAGACAAACGAACGCATAGAGGAAATTA
TAAGAACAACCGGCAAAGAGAATGCCAAGTATCTGATCGAGAAAATCAA
GCTGCACGACATGCAAGAAGGCAAGTGCCTGTACTCTCTGGAAGCTATCC
CACTCGAAGATCTGCTGAATAATCCATTCAATTACGAGGTGGACCACATC
ATCCCTAGATCCGTAAGCTTTGACAATTCCTTCAATAACAAAGTTCTGGTT
AAACAGGAGGAAAATTCTAAAAAAGGGAACCGGACCCCGTTCCAGTACC
TGAGCTCCAGTGACAGCAAGATTAGCTACGAGACTTTTAAGAAACATATT
CTGAATCTGGCCAAAGGCAAAGGCAGGATCAGCAAGACCAAGAAGGAGT
ACCTCCTCGAAGAACGCGACATTAACAGATTTAGTGTGCAGAAAGATTTC
ATCAACCGAAACCTTGTCGATACTCGGTACGCCACGAGAGGCCTGATGAA
TCTCCTCAGGAGCTACTTCCGCGTCAATAATCTGGACGTTAAAGTCAAGA
GCATAAATGGGGGATTCACCAGCTTTCTGAGGAGAAAGTGGAAGTTTAAG
AAGGAACGAAACAAAGGATACAAGCACCATGCTGAGGATGCTTTGATCA
TCGCTAACGCGGACTTTATCTTTAAGGAATGGAAAAAGCTGGATAAGGCA
AAGAAAGTGATGGAAAACCAGATGTTCGAGGAGAAGCAGGCAGAGTCAA
TGCCTGAGATCGAGACAGAGCAGGAATACAAGGAAATTTTCATCACCCCT
CATCAGATTAAACACATAAAGGACTTCAAAGACTATAAATACTCTCATAG
GGTGGACAAAAAACCCAATCGCAAGCTCATTAATGACACCCTGTACTCAA
CACGGAAGGATGATAAAGGTAATACCTTGATTGTGAATAATCTTAATGGA
TTGTATGACAAAGATAACGACAAGCTCAAGAAGCTGATCAACAAGTCTCC
AGAGAAGCTCCTTATGTATCACCACGACCCACAGACTTATCAGAAATTGA
AACTGATCATGGAGCAATACGGGGATGAGAAGAACCCACTCTACAAATAT
TATGAGGAAACAGGTAATTACCTGACCAAGTACTCCAAGAAGGATAACG
GACCAGTGATCAAAAAGATAAAGTACTATGGCAACAAACTTAATGCGCAT
TTGGACATAACTGACGATTACCCCAATTCTCGAAACAAGGTTGTGAAGCT
CTCCCTGAAGCCTTATAGATTTGACGTGTACCTGGATAATGGGGTTTATAA
ATTCGTCACCGTGAAAAATCTGGACGTGATCAAAAAGGAGAACTATTATG
AAGTAAACTCAAAGTGCTATGAGGAGGCGAAGAAGCTGAAGAAGATCTC
CAATCAGGCCGAGTTCATCGCTTCCTTCTATAAGAACGATCTCATCAAGAT
CAATGGAGAGCTTTATCGCGTCATTGGTGTGAACAATGACTTGCTGAACA
GGATCGAAGTCAATATGATAGACATTACCTACCGGGAGTATCTCGAAAAC
ATGAATGATAAACGGCCGCCTCACATCATCAAGACAATCGCATCTAAAAC
TCAGTCAATAAAAAAGTACTCTACCGATATCCTGGGGAATCTCTATGAAG
TGAAGTCAAAGAAGCACCCACAAATCATTAAAAAAGGTGGATCCCCCAA
GAAGAAGAGGAAAGTCTCGAGCGACTACAAAGACCATGACGGTGATTAT
AAAGATCATGACATCGATTACAAGGATGACGATGACAAGTCTGGTGGTTC
TACTAATCTGTCAGATATTATTGAAAAGGAGACCGGTAAGCAACTGGTTA
TCCAGGAATCCATCCTCATGCTCCCAGAGGAGGTGGAAGAAGTCATTGGG
AACAAGCCGGAAAGCGATATACTCGTGCACACCGCCTACGACGAGAGCA
CCGACGAGAATGTCATGCTTCTGACTAGCGACGCCCCTGAATACAAGCCT
TGGGCTCTGGTCATACAGGATAGCAACGGTGAGAACAAGATTAAGATGCT
CTCTGGTGGTTCTCCCAAGAAGAAGAGGAAAGTCTAA

1×GCN4-xCas9

ATGGGACCCAAGAAAAAACGCAAGGTGGAAGATCCTAAGAAAAAGCGG
AAAGTGGACGGCATTGGTAGTGGGAGCAACGGCAGCAGCGGAGAGGAAC
TGCTGAGCAAGAACTACCACCTGGAAAACGAGGTGGCCAGACTGAAGAA
AGGCTCTGGCTCTGGCGGAAGCGGTTCTGGCGGATCAGGATCTGGAAGTG
GCGGCGACAAGAAGTACTCCATTGGGCTCGCTATCGGCACAAACAGCGTC
GGCTGGGCCGTCATTACGGACGAGTACAAGGTGCCGAGCAAAAAATTCA
AAGTTCTGGGCAATACCGATCGCCACAGCATAAAGAAGAACCTCATTGGC
GCCCTCCTGTTCGACTCCGGGGAGACGGCCGAAGCCACGCGGCTCAAAAG
AACAGCACGGCGCAGATATACCCGCAGAAAGAATCGGATCTGCTACCTGC
AGGAGATCTTTAGTAATGAGATGGCTAAGGTGGATGACTCTTTCTTCCATA
GGCTGGAGGAGTCCTTTTTGGTGGAGGAGGATAAAAAGCACGAGCGCCA
CCCAATCTTTGGCAATATCGTGGACGAGGTGGCGTACCATGAAAAGTACC
CAACCATATATCATCTGAGGAAGAAGCTTGTAGACAGTACTGATAAGGCT
GACTTGCGGTTGATCTATCTCGCGCTGGCGCATATGATCAAATTTCGGGGA
CACTTCCTCATCGAGGGGGACCTGAACCCAGACAACAGCGATGTCGACAA
ACTCTTTATCCAACTGGTTCAGACTTACAATCAGCTTTTCGAAGAGAACCC
GATCAACGCATCCGGAGTTGACGCCAAAGCAATCCTGAGCGCTAGGCTGT
CCAAATCCCGGCGGCTCGAAAACCTCATCGCACAGCTCCCTGGGGAGAAG
AAGAACGGCCTGTTTGGTAATCTTATCGCCCTGTCCCTCGGGCTGACCCCC
AACTTTAAATCTAACTTCGACCTGGCCGAAGATACCAAGCTTCAACTGAG
CAAAGACACCTACGATGATGATCTCGACAATCTGCTGGCCCAGATCGGCG
ACCAGTACGCAGACCTTTTTTTGGCGGCAAAGAACCTGTCAGACGCCATT
CTGCTGAGTGATATTCTGCGAGTGAACACGGAGATCACCAAAGCTCCGCT
GAGCGCTAGTATGATCAAGCTCTATGATGAGCACCACCAAGACTTGACTT
TGCTGAAGGCCCTTGTCAGACAGCAACTGCCTGAGAAGTACAAGGAAATT
TTCTTCGATCAGTCTAAAAATGGCTACGCCGGATACATTGACGGCGGAGC
AAGCCAGGAGGAATTTTACAAATTTATTAAGCCCATCTTGGAAAAAATGG
ACGGCACCGAGGAGCTGCTGGTAAAGCTTAACAGAGAAGATCTGTTGCGC
AAACAGCGCACTTTCGACAATGGAATCATCCCCCACCAGATTCACCTGGG
CGAACTGCACGCTATCCTCAGGCGGCAAGAGGATTTCTACCCCTTTTTGAA
AGATAACAGGGAAAAGATTGAGAAAATCCTCACATTTCGGATACCCTACT
ATGTAGGCCCCCTCGCCCGGGGAAATTCCAGATTCGCGTGGATGACTCGC
AAATCAGAAGAGACCATCACTCCCTGGAACTTCGAGAAAGTCGTGGATAA
GGGGGCCTCTGCCCAGTCCTTCATCGAAAGGATGACTAACTTTGATAAAA
ATCTGCCTAACGAAAAGGTGCTTCCTAAACACTCTCTGCTGTACGAGTACT
TCACAGTTTATAACGAGCTCACCAAGGTCAAATACGTCACAGAAGGGATG
AGAAAGCCAGCATTCCTGTCTGGAGATCAGAAGAAAGCTATTGTGGACCT
CCTCTTCAAGACGAACCGGAAAGTTACCGTGAAACAGCTCAAAGAAGACT
ATTTCAAAAAGATTGAATGTTTCGACTCTGTTGAAATCAGCGGAGTGGAG
GATCGCTTCAACGCATCCCTGGGAACGTATCACGATCTCCTGAAAATCATT
AAAGACAAGGACTTCCTGGACAATGAGGAGAACGAGGACATTCTTGAGG
ACATTGTCCTCACCCTTACGTTGTTTGAAGATAGGGAGATGATTGAAGAA
CGCTTGAAAACTTACGCTCATCTCTTCGACGACAAAGTCATGAAGCAGCT

CAAGAGGCGCCGATATACAGGATGGGGGCGGCTGTCAAGAAAACTGATC
AATGGGATCCGAGACAAGCAGAGTGGAAAGACAATCCTGGATTTTCTTAA
GTCCGATGGATTTGCCAACCGGAACTTCATTCAGTTGATCCATGATGACTC
TCTCACCTTTAAGGAGGACATCCAGAAAGCACAAGTTTCTGGCCAGGGGG
ACAGTCTTCACGAGCACATCGCTAATCTTGCAGGTAGCCCAGCTATCAAA
AAGGGAATACTGCAGACCGTTAAGGTCGTGGATGAACTCGTCAAAGTAAT
GGGAAGGCATAAGCCCGAGAATATCGTTATCGAGATGGCCCGAGAGAAC
CAAACCACCCAGAAGGGACAGAAGAACAGTAGGGAAAGGATGAAGAGG
ATTGAAGAGGGTATAAAAGAACTGGGGTCCCAAATCCTTAAGGAACACCC
AGTTGAAAACACCCAGCTTCAGAATGAGAAGCTCTACCTGTACTACCTGC
AGAACGGCAGGGACATGTACGTGGATCAGGAACTGGACATCAATCGGCT
CTCCGACTACGACGTGGATCATATCGTGCCCCAGTCTTTTCTCAAAGATGA
TTCTATTGATAATAAAGTGTTGACAAGATCCGATAAAAACAGAGGGAAGA
GTGATAACGTCCCCTCAGAAGAAGTTGTCAAGAAAATGAAAAATTATTGG
CGGCAGCTGCTGAACGCCAAACTGATCACACAACGGAAGTTCGATAATCT
GACTAAGGCTGAACGAGGTGGCCTGTCTGAGTTGGATAAAGCCGGTTTCA
TCAAAAGGCAGCTTGTTGAGACACGCCAGATCACCAAGCACGTGGCCCAA
ATTCTCGATTCACGCATGAACACCAAGTACGATGAAAATGACAAACTGAT
TCGAGAGGTGAAAGTTATTACTCTGAAGTCTAAGCTGGTCTCAGATTTCAG
AAAGGACTTTCAGTTTTATAAGGTGAGAGAGATCAACAATTACCACCATG
CGCATGATGCCTACCTGAATGCAGTGGTAGGCACTGCACTTATCAAAAAA
TATCCCAAGCTTGAATCTGAATTTGTTTACGGAGACTATAAAGTGTACGAT
GTTAGGAAAATGATCGCAAAGTCTGAGCAGGAAATAGGCAAGGCCACCG
CTAAGTACTTCTTTTACAGCAATATTATGAATTTTTTCAAGACCGAGATTA
CACTGGCCAATGGAGAGATTCGGAAGCGACCACTTATCGAAACAAACGG
AGAAACAGGAGAAATCGTGTGGGACAAGGGTAGGGATTTCGCGACAGTC
CGGAAGGTCCTGTCCATGCCGCAGGTGAACATCGTTAAAAAGACCGAAGT
ACAGACCGGAGGCTTCTCCAAGGAAAGTATCCTCCCGAAAAGGAACAGC
GACAAGCTGATCGCACGCAAAAAAGATTGGGACCCCAAGAAATACGGCG
GATTCGATTCTCCTACAGTCGCTTACAGTGTACTGGTTGTGGCTAAAGTGG
AGAAAGGGAAGTCTAAAAAACTCAAAAGCGTCAAGGAACTGCTGGGCAT
CACAATCATGGAGCGATCAAGCTTCGAAAAAAACCCCATCGACTTTCTCG
AGGCGAAAGGATATAAAGAGGTCAAAAAAGACCTCATCATTAAGCTTCCC
AAGTACTCTCTCTTTGAGCTTGAAAACGGCCGGAAACGAATGCTCGCTAG
TGCGGGCGTGCTGCAGAAAGGTAACGAGCTGGCACTGCCCTCTAAATACG
TTAATTTCTTGTATCTGGCCAGCCACTATGAAAAGCTCAAAGGGTCTCCCG
AAGATAATGAGCAGAAGCAGCTGTTCGTGGAACAACACAAACACTACCTT
GATGAGATCATCGAGCAAATAAGCGAATTCTCCAAAAGAGTGATCCTCGC
CGACGCTAACCTCGATAAGGTGCTTTCTGCTTACAATAAGCACAGGGATA
AGCCCATCAGGGAGCAGGCAGAAAACATTATCCACTTGTTTACTCTGACC
AACTTGGGCGCGCCTGCAGCCTTCAAGTACTTCGACACTACCATAGACAG
AAAGCGGTACACCTCTACAAAGGAGGTCCTGGACGCCACACTGATTCATC
AGTCAATTACGGGGCTCTATGAAACAAGAATCGACCTCTCTCAGCTCGGT
GGAGACGAGGGAGCTGATAAGCGCACCGCCGATGGTTCCGAGTTCGAAA

GCCCCAAGAAGAAGAGGAAAGTCTAA

pCMV-ABE-scFv

ATGCACCAAAGAAGAAGCGGAAAGTCTCTGAAGTCGAGTTTAGCCACGA
GTATTGGATGAGGCACGCACTGACCCTGGCAAAGCGAGCATGGGATGAA
AGAGAAGTCCCCGTGGGCGCCGTGCTGGTGCACAACAATAGAGTGATCGG
AGAGGGATGGAACAGGCCAATCGGCCGCCACGACCCTACCGCACACGCA
GAGATCATGGCACTGAGGCAGGGAGGCCTGGTCATGCAGAATTACCGCCT
GATCGATGCCACCCTGTATGTGACACTGGAGCCATGCGTGATGTGCGCAG
GAGCAATGATCCACAGCAGGATCGGAAGAGTGGTGTTCGGAGCACGGGA
CGCCAAGACCGGCGCAGCAGGCTCCCTGATGGATGTGCTGCACCACCCCG
GCATGAACCACCGGGTGGAGATCACAGAGGGAATCCTGGCAGACGAGTG
CGCCGCCCTGCTGAGCGATTTCTTTAGAATGCGGAGACAGGAGATCAAGG
CCCAGAAGAAGGCACAGAGCTCCACCGACTCTGGAGGATCTAGCGGAGG
ATCCTCTGGAAGCGAGACACCAGGCACAAGCGAGTCCGCCACACCAGAG
AGCTCCGGCGGCTCCTCCGGAGGATCCTCTGAGGTGGAGTTTTCCCACGA
GTACTGGATGAGACATGCCCTGACCCTGGCCAAGAGGGCACGCGATGAG
AGGGAGGTGCCTGTGGGAGCCGTGCTGGTGCTGAACAATAGAGTGATCGG
CGAGGGCTGGAACAGAGCCATCGGCCTGCACGACCCAACAGCCCATGCC
GAAATTATGGCCCTGAGACAGGGCGGCCTGGTCATGCAGAACTACAGACT
GATTGACGCCACCCTGTACGTGACATTCGAGCCTTGCGTGATGTGCGCCG
GCGCCATGATCCACTCTAGGATCGGCCGCGTGGTGTTTGGCGTGAGGAAC
GCAAAAACCGGCGCCGCAGGCTCCCTGATGGACGTGCTGCACTACCCCGG
CATGAATCACCGCGTCGAAATTACCGAGGGAATCCTGGCAGATGAATGTG
CCGCCCTGCTGTGCTATTTCTTTCGGATGCCTAGACAGGTGTTCAATGCTC
AGAAGAAGGCCCAGAGCTCCACCGACTCCGGAGGATCTAGCGGAGGCTC
CTCTGGCTCTGAGACACCTGGCACAAGCGAGAGCGCAACACCTGAAAGCT
CCGGAGGATCTAGCGGAGGCTCCTCTGGTGGTTCTTCTGGTGGTTCTAGCG
GGGGCAGCAGCGGGGGGTCAATGGGTCCCGACATCGTGATGACCCAGAG
CCCCAGCAGCCTGAGCGCCAGCGTGGGCGACCGCGTGACCATCACCTGCC
GCAGCAGCACCGGCGCCGTGACCACCAGCAACTACGCCAGCTGGGTGCA
GGAGAAGCCCGGCAAGCTGTTCAAGGGCCTGATCGGCGGCACCAACAAC
CGCGCCCCCGGCGTGCCCAGCCGCTTCAGCGGCAGCCTGATCGGCGACAA
GGCCACCCTGACCATCAGCAGCCTGCAGCCCGAGGACTTCGCCACCTACT
TCTGCGCCCTGTGGTACAGCAACCACTGGGTGTTCGGCCAGGGCACCAAG
GTGGAGCTGAAGCGCGGCGGCGGCGGCAGCGGCGGCGGCGGCAGCGGCG
GCGGCGGCAGCAGCGGCGGCGGCAGCGAGGTGAAGCTGCTGGAGAGCGG
CGGCGGCCTGGTGCAGCCCGGCGGCAGCCTGAAGCTGAGCTGCGCCGTGA
GCGGCTTCAGCCTGACCGACTACGGCGTGAACTGGGTGCGCCAGGCCCCC
GGCCGCGGCCTGGAGTGGATCGGCGTGATCTGGGGCGACGGCATCACCGA
CTACAACAGCGCCCTGAAGGACCGCTTCATCATCAGCAAGGACAACGGCA
AGAACACCGTGTACCTGCAGATGAGCAAGGTGCGCAGCGACGACACCGC
CCTGTACTACTGCGTGACCGGCCTGTTCGACTACTGGGGCCAGGGCACCC
TGGTGACCGTGAGCAGCTACCCATACGATGTTCCAGATTACGCTGGTTCTG

GCGGCTCAAAAAGAACCGCCGACGGCAGCGAATTCGAGCCCAAGAAGAA
GAGGAAAGTCTAA

pVax-PAH-WT

ATGTCCACTGCGGTCCTGGAAAACCCAGGCTTGGGCAGGAAACTCTCTGA
CTTTGGACAGGAAACAAGCTATATTGAAGACAACTGCAATCAAAATGGTG
CCATATCACTGATCTTCTCACTCAAAGAAGAAGTTGGTGCATTGGCCAAA
GTATTGCGCTTATTTGAGGAGAATGATGTAAACCTGACCCACATTGAATCT
AGACCTTCTCGTTTAAAGAAAGATGAGTATGAATTTTTCACCCATTTGGAT
AAACGTAGCCTGCCTGCTCTGACAAACATCATCAAGATCTTGAGGCATGA
CATTGGTGCCACTGTCCATGAGCTTTCACGAGATAAGAAGAAAGACACAG
TGCCCTGGTTCCCAAGAACCATTCAAGAGCTGGACAGATTTGCCAATCAG
ATTCTCAGCTATGGAGCGGAACTGGATGCTGACCACCCTGGTTTTAAAGA
TCCTGTGTACCGTGCAAGACGGAAGCAGTTTGCTGACATTGCCTACAACT
ACCGCCATGGGCAGCCCATCCCTCGAGTGGAATACATGGAGGAAGAAAA
GAAAACATGGGGCACAGTGTTCAAGACTCTGAAGTCCTTGTATAAAACCC
ATGCTTGCTATGAGTACAATCACATTTTTCCACTTCTTGAAAAGTACTGTG
GCTTCCATGAAGATAACATTCCCCAGCTGGAAGACGTTTCTCAATTCCTGC
AGACTTGCACTGGTTTCCGCCTCCGACCTGTGGCTGGCCTGCTTTCCTCTC
GGGATTTCTTGGGTGGCCTGGCCTTCCGAGTCTTC**C**ACTGCACACAGTACA
TCAGACATGGATCCAAGCCCATGTATACCCCCGAACCTGACATCTGCCAT
GAGCTGTTGGGACATGTGCCCTTGTTTTCAGATCGCAGCTTTGCCCAGTTT
TCCCAGGAAATTGGCCTTGCCTCTCTGGGTGCACCTGATGAATACATTGAA
AAGCTCGCCACAATTTACTGGTTTACTGTGGAGTTTGGGCTCTGCAAACAA
GGAGACTCCATAAAGGCATATGGTGCTGGGCTCCTGTCATCCTTTGGTGA
ATTACAGTACTGCTTATCAGAGAAGCCAAAGCTTCTCCCCCTGGAGCTGG
AGAAGACAGCCATCCAAAATTACACTGTCACGGAGTTCCAGCCCCTGTAT
TACGTGGCAGAGAGTTTTAATGATGCCAAGGAGAAAGTAAGGAACTTTGC
TGCCACAATACCTCGGCCCTTCTCAGTTCGCTACGACCCATACACCCAAAG
GATTGAGGTCTTGGACAATACCCAGCAGCTTAAGATTTTGGCTGATTCCAT
TAACAGTGAAATTGGAATCCTTTGCAGTGCCCTCCAGAAAATAAAGTAA

pVax-PAH-H264Y

ATGTCCACTGCGGTCCTGGAAAACCCAGGCTTGGGCAGGAAACTCTCTGA
CTTTGGACAGGAAACAAGCTATATTGAAGACAACTGCAATCAAAATGGTG
CCATATCACTGATCTTCTCACTCAAAGAAGAAGTTGGTGCATTGGCCAAA
GTATTGCGCTTATTTGAGGAGAATGATGTAAACCTGACCCACATTGAATCT
AGACCTTCTCGTTTAAAGAAAGATGAGTATGAATTTTTCACCCATTTGGAT
AAACGTAGCCTGCCTGCTCTGACAAACATCATCAAGATCTTGAGGCATGA
CATTGGTGCCACTGTCCATGAGCTTTCACGAGATAAGAAGAAAGACACAG
TGCCCTGGTTCCCAAGAACCATTCAAGAGCTGGACAGATTTGCCAATCAG
ATTCTCAGCTATGGAGCGGAACTGGATGCTGACCACCCTGGTTTTAAAGA
TCCTGTGTACCGTGCAAGACGGAAGCAGTTTGCTGACATTGCCTACAACT
ACCGCCATGGGCAGCCCATCCCTCGAGTGGAATACATGGAGGAAGAAAA

GAAAACATGGGGCACAGTGTTCAAGACTCTGAAGTCCTTGTATAAAACCC
ATGCTTGCTATGAGTACAATCACATTTTTCCACTTCTTGAAAAGTACTGTG
GCTTCCATGAAGATAACATTCCCCAGCTGGAAGACGTTTCTCAATTCCTGC
AGACTTGCACTGGTTTCCGCCTCCGACCTGTGGCTGGCCTGCTTTCCTCTC
GGGATTTCTTGGGTGGCCTGGCCTTCCGAGTCTTC**T**ACTGCACACAGTACA
TCAGACATGGATCCAAGCCCATGTATACCCCCGAACCTGACATCTGCCAT
GAGCTGTTGGGACATGTGCCCTTGTTTTCAGATCGCAGCTTTGCCCAGTTT
TCCCAGGAAATTGGCCTTGCCTCTCTGGGTGCACCTGATGAATACATTGAA
AAGCTCGCCACAATTTACTGGTTTACTGTGGAGTTTGGGCTCTGCAAACAA
GGAGACTCCATAAAGGCATATGGTGCTGGGCTCCTGTCATCCTTTGGTGA
ATTACAGTACTGCTTATCAGAGAAGCCAAAGCTTCTCCCCCTGGAGCTGG
AGAAGACAGCCATCCAAAATTACACTGTCACGGAGTTCCAGCCCCTGTAT
TACGTGGCAGAGAGTTTTAATGATGCCAAGGAGAAAGTAAGGAACTTTGC
TGCCACAATACCTCGGCCCTTCTCAGTTCGCTACGACCCATACACCCAAAG
GATTGAGGTCTTGGACAATACCCAGCAGCTTAAGATTTTGGCTGATTCCAT
TAACAGTGAAATTGGAATCCTTTGCAGTGCCCTCCAGAAAATAAAGTAA
