# Supplementary material for: A universal strategy for AAV delivery of base editors to correct genetic point mutations in neonatal PKU mice
Source: Mol Ther Methods Clin Dev. 2022 Jan 7;24:230–40. doi: 10.1016/j.omtm.2022.01.001 (PMC8803597; doi:10.1016/j.omtm.2022.01.001)
Supplement: Document S2. Article plus supplemental information [file mmc2.pdf]

Molecular Therapy
Methods & Clinical Development
Original Article

A universal strategy for AAV delivery of base editors to correct genetic point mutations in neonatal PKU mice

Lifang Zhou,[1,2] Jing Su,[1,2] Jie Long,[1] Rui Tao,[1] Wenling Tang,[1] Fengming Qin,[1] Nan Liu,[1] Yanhong Wang,[1] Yaoge Jiao,[1] Yun Hu,[1] Lurong Jiang,[1] Li Li,[1] Yang Yang,[1] and Shaohua Yao[1]

[1]Laboratory of Biotherapy, National Key Laboratory of Biotherapy, Cancer Center, West China Hospital, Sichuan University, Renmin Nanlu 17, Chengdu 610041, Sichuan, China

Base editing tools enabled efficient conversion of C:G or A:T base pairs to T:A or G:C, which are especially powerful for targeting monogenic lesions. However, *in vivo* correction of disease-causing mutations is still less efficient because of the large size of base editors. Here, we designed a dual adeno-associated virus (AAV) strategy for *in vivo* delivery of base editors, in which deaminases were linked to Cas9 through the interaction of GCN4 peptide and its single chain variable fragment (scFv) antibody. We found that one or two copies of GCN4 peptide were enough for the assembly of base editors and produced robust targeted editing. By optimization of single-guide RNAs (sgRNAs) that target phenylketonuria (PKU) mutation, we were able to achieve up to 27.7% correction *in vitro*. *In vivo* delivery of this dual AAV base editing system resulted in efficient correction of PKU-related mutation in neonatal mice and subsequent rescue of hyperphenylalaninemia-associated syndromes. Considering the similarity between Cas9 proteins from different organisms, our delivery strategy will be compatible with other Cas9-derived base editors.

INTRODUCTION

Clustered regularly interspaced short palindromic repeats (CRISPR)/CRISPR-associated protein 9 (Cas9) is an immune system in bacteria and archaea that targets foreign nucleic acids.[1,2] It quickly revolutionized the field of genome engineering as pioneering work demonstrated the programmability and efficiency of this system, especially type II CRISPR-Cas9, in eukaryotic cells.[3,4] Under the guidance of a single-guide RNA (sgRNA), Cas9 binds to target DNA and produces double-strand breaks (DSBs), which are then repaired by non-homologous end joining (NHEJ) or homology-directed repair (HDR) mechanisms in eukaryotic cells.[1] In addition to inducing DSBs, Cas9 can also be engineered to bind DNA and recruit DNA regulators or modifiers to induce local DNA manipulation.[5–10]

Structurally, when bound to target DNA, Cas9 disassociates a small fragment of the non-target strand (NTS) from the target strand (TS),[11] which is exposed outside the Cas9/DNA/sgRNA complex and can therefore serve as substrate for enzymes with single-stranded DNA (ssDNA) preference.[11] Based on this notion, a base editing technology was developed to convert a single base within the exposed NTS by the action of cytosine or adenosine deaminases coupled to the Cas9 complex.[9,10] This technology enables efficient and irreversible conversion of C:G or A:T base pairs to T:A or G:C and is particularly effective in the treatment of genetic disorders caused by single point mutations.

Point mutations are the major genetic lesions in a panel of inherited diseases, including phenylketonuria (PKU).[12] PKU is an autosomal recessive genetic disorder caused by a loss-of-function mutation of the phenylalanine hydroxylase (*Pah*) gene responsible for the conversion of phenylalanine to tyrosine.[13] PAH deficiency results in the accumulation of phenylalanine and its metabolites, leading to developmental delays and mental retardation. Approximately 60% of these point mutations found in PKU patients are base transitions,[14] i.e., base substitutions between pyrimidines or purines, all of which can theoretically be corrected with base editing tools. Despite the remarkable success of base editing technology in *in vitro* studies, *in vivo* application remains challenging. Current base editors range in size from 4.2 kb to 5.2 kb, depending on the Cas9s and deaminases used, approaching or exceeding the packaging limitations of AAV (~4.5 kb), which is one of the most efficient *in vivo* delivery vectors.[15] Recently, the AAV delivery of split base editors through the use of an intein splicing system has been developed to treat an adult PKU mouse model that phenocopies human patients with hyperphenylalaninemia, hypopigmentation, and mental retardation.[16] To the best of our knowledge, intein-based dual AAV base editors have not been

Received 15 June 2021; accepted 5 January 2022;
https://doi.org/10.1016/j.omtm.2022.01.001.

[2]These authors contributed equally

Correspondence: Shaohua Yao, PhD, Laboratory of Biotherapy, National Key Laboratory of Biotherapy, Cancer Center, West China Hospital, Sichuan University, Renmin Nanlu 17, Chengdu 610041, Sichuan, China.
E-mail: shaohuayao@scu.edu.cn
Correspondence: Yang Yang, PhD, Laboratory of Biotherapy, National Key Laboratory of Biotherapy, Cancer Center, West China Hospital, Sichuan University, Renmin Nanlu 17, Chengdu 610041, Sichuan, China.
E-mail: yang2012@scu.edu.cn

 © 2022 The Authors.
This is an open access article under the CC BY license (http://creativecommons.org/licenses/by/4.0/).

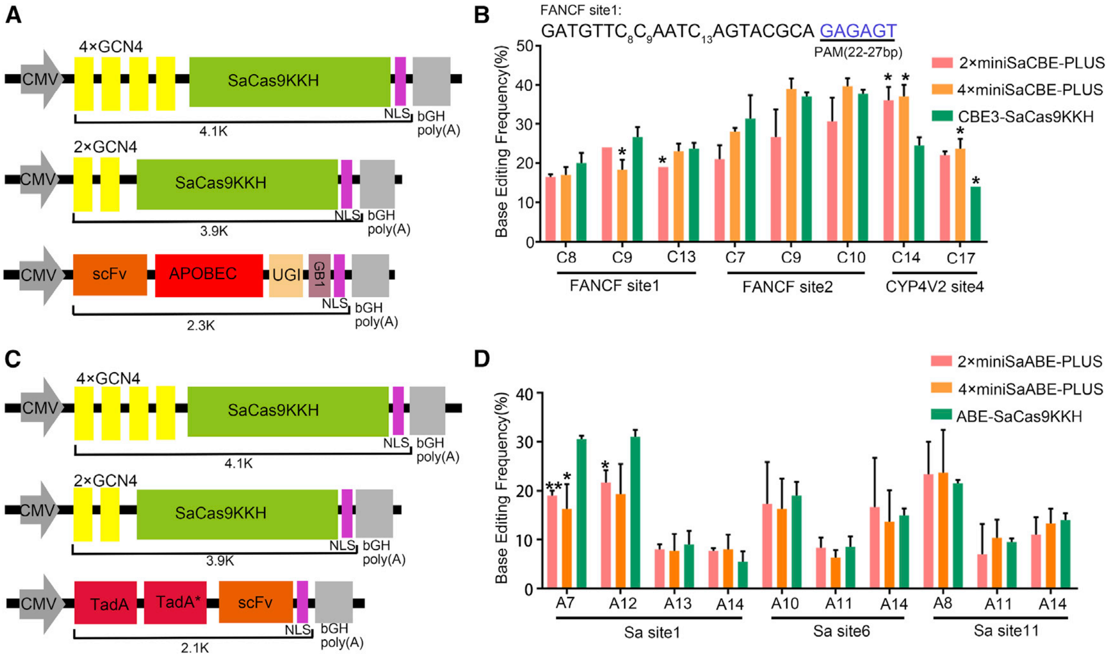

Figure 1. Design and characterization of miniSaBE-PLUS editing system

(A) Schematic diagram showing the expression cassettes of miniSaCBE-PLUS plasmids, pCMV-4×SaCas9KKH, pCMV-2×SaCas9KKH, and pCMV-scFv-CBE. (B) Cytosine base editing of 2×miniSaCBE-PLUS, 4×miniSaCBE-PLUS, and CBE3-SaCas9KKH at three genomic loci in HEK 293T cells. FANCF site1 and FANCF site2 are two different target sites in FANCF locus. The sequence of FANCF site1 is listed above. PAM is shown in blue and counted as 22–26 (n = 3 independent experiments). Values represent mean ± SD. Asterisks indicate statistically significant differences in editing efficiencies observed between CBE-SaCas9KKH and 4×miniSaCBE-PLUS or 2×miniSaCBE-PLUS at each site. (C) Schematic diagram showing the expression cassettes of miniSaCBE-PLUS plasmids, pCMV-4×SaCas9KKH, pCMV-2×SaCas9KKH, and pCMV-scFv-ABE. (D) Adenine base editing of 2×miniSaABE-PLUS, 4×miniSaABE-PLUS, and ABE-SaCas9KKH at three genomic loci in HEK 293T cells (n = 3 independent experiments). Values represent mean ± SD. Asterisks indicate statistically significant differences in editing efficiencies observed between ABE-SaCas9KKH and 4×miniSaABE-PLUS or 2×miniSaABE-PLUS at each site. Abbreviations are as follows: CMV, human cytomegalovirus promoter; UGI, uracil DNA glycosylase inhibitor; NLS, nuclear localization signal; GB1, G protein B1 domain.

tested in neonatal animals. However, treatment at an early stage is required to prevent the secondary effects of toxic metabolites in PKU patients.[12]

Here, we designed a dual-AAV strategy for *in vivo* delivery of base editors in which deaminases are linked to Cas9 through the interaction of the GCN4 peptide and its scFv.[17,18] Previous studies have shown that 10×GCN4-tagged Cas9 proteins successfully recruit cytosine deaminase to assemble a functional base editor, BE-PLUS [short for programming larger C to U (T) scope].[17] We found that one or two copies of the GCN4 peptide were sufficient to assemble base editors and produce robust targeted editing (miniBE-PLUS) with an efficiency comparable to that produced by the corresponding full-length base editors. Based on this finding, we designed a dual-AAV expression system in which one AAV expresses the 2×GCN4-tagged SaCas9KKH nickase (D10A) and the other expresses sgRNA and cytosine deaminase (miniSaCBE-PLUS). We used the resulting AAVs to treat a PKU (B6.BTBR-*Pah*^{enu2}) mouse model[19] at the neonatal stage and achieved genetically corrected therapeutic levels leading to normalization of phenylalanine (Phe) metabolism. Therefore, these data demonstrate the potential of our miniBE-PLUS system for the early treatment of genetic disorders.

RESULTS

Design and characterization of AAV-compatible miniBE-PLUS

In the BE-PLUS system, 10 copies of 19-aa GCN4 were fused to the spCas9 protein to recruit the cytosine deaminase fused to an scFV that binds specifically to GCN4. Although efficient, BE-PLUS was larger than 5.6 kb, exceeding the packaging capacity of AAV. Here, to facilitate AAV delivery, we designed minimal BE-PLUS editors based on a smaller Cas9, SaCas9. We attached two or four copies of GCN4 to the N-terminus of the SaCas9KKH D10A nickase and then coupled it to the scFv-APOBEC1 fusion protein to form 4×miniSaCBE-PLUS or 2×miniSaCBE-PLUS, respectively (Figure 1A). APOBEC1 is a strong cytosine deaminase widely used in the base editing system. Each GCN4 peptide was linked to a $(GSGSG)_5$ flexible linker. Transfection of these miniSaCBE-PLUS plasmids with SaCas9 sgRNAs produced significant C to T editing at the target genomic loci. The editing activity of miniSaCBE-PLUS on all three targets was comparable to that of full-length BE3-SaCas9KKH (Figure 1B). In addition, we fused a pair of tRNA adenosine deaminases (TadAs), one of which was mutated to acquire the deamination activity of adenines in DNA, to the N-terminus of scFv and coupled it to 2×GCN4-SaCasKKH or 4×GCN4-SaCasKKH to obtain miniSaABE-PLUS (Figure 1C). The editing activity of miniSaABE-PLUS

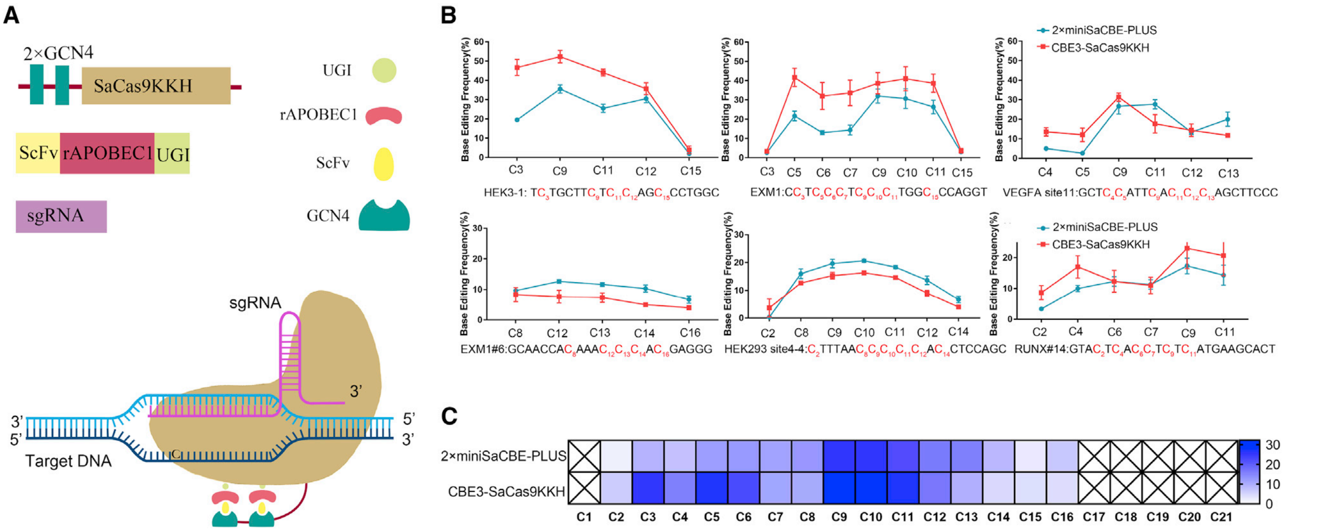

Figure 2. Editing windows of 2×miniSaCBE-PLUS

(A) Schematic diagram showing the elements of 2×miniSaCBE-PLUS and the model of its action. (B) Comparison of the editing window of 2×miniSaCBE-PLUS and CBE3-SaCas9KKH at six genomic loci of HEK 293T cells (n = 3 independent experiments). Values represent mean ± SD. (C) Heatmap summarizing the editing efficiency of each cytosine in the editing window. The average C-to-T editing rate of indicated position was calculated from six target sites shown in (B) (n = 3 independent experiments). Values represent mean ± SD.

on all three targets was similar to that of ABE-SaCas9KKH (Figure 1D). As SpCas9 and its variants are more widely used, we also designed an AAV-compatible SpCas9 base editor in which a copy of GCN4 was fused to the N-terminus of SpCas9 in the version of xCas9 (Figures S1A and S1C). The resulting 1×minixCBE-PLUS showed editing activity comparable to that of full-length BE3-xCas9, while 1×minixABE-PLUS was slightly less efficient than full-length ABE-xCas9 (Figure S1D).

As 2×miniSaCBE-PLUS is smaller than 4×miniSaCBE-PLUS but with similar editing efficiency, we chose 2×miniSaCBE-PLUS for further characterization. First, we determined the editing window of 2×miniSaCBE-PLUS with a series of targets containing multiple Cs in the putative editing window. As shown in Figure 2, 2×miniSaCBE-PLUS had an editing window similar to that of full-length CBE3-SaCas9KKH, ranging from 2 to 17 (counting NNNRRT PAM as 22–26).

Targeting of mutant *Pah*

Next, we tested whether our 2×miniSaCBE-PLUS editor could correct genetic disease by using PKU as a disease model (B6.BTBR-*Pah*^{enu2}). Similar to human patients, B6.BTBR-*Pah*^{enu2} mice are genetically recessive and carry a missense mutation (c.835T→C, F263S) in the *Pah* gene, which can be rescued with the cytosine base editor. As a first step in testing, we constructed a plasmid target harboring the mutant fragment for screening editing strategies (PKU target, Ptarget), as plasmids have been reported to serve as substrates for base editors when co-transfected into cells. A previous study has identified an sgRNA that efficiently guides CBE3-SaCas9KKH to the PKU mutant site to correct the point mutation. This sgRNA has a 21-nt protospacer region and uses a protospacer adjacent motif (PAM) at 9 nt downstream of the PKU mutation (PKU-sgRNA21) (Figure 3A). Consistent with previous reports, we found that co-transfection of this sgRNA with CBE3-SaCas9KKH and Ptarget into 293T cells resulted in significant editing of the target base [C13T, TCC to TTC or TTT, Ser (S) 263 Phe (F)] (Figure 3B).

We then tested whether PKU-sgRNA21 was also functional when coupled to 2×miniSaCBE-PLUS. As expected, co-transfection of PKU-sgRNA21 with 2×miniSaCBE-PLUS and Ptarget into 293T cells produced robust editing of the target base with an efficiency comparable to that of CBE3-SaCas9KKH (Figure 3B).

We note that there are additional cytosines surrounding the target cytosine, all of which have been considerably edited. As shown in Figure 3A, editing those non-targeted cytosines (bystanders) resulted in synonymous [C5T, TTC to TTT, F260F; C11T, GTC to GTT, Val (V) 262 Val], missense [C15T, CAC to TAC/TAT, His (H) 264 Tyr (Y)], or non-sense [C6T, CGA to TGA, Arg (R) 260 stop codon (*)] mutations (Figure 3A). To determine whether missense editing (H264Y) would silence PAH activity, we constructed two PAH expression vectors, one expressing wild-type PAH (pVax-WT-PAH) and the other expressing PAH H264Y (pVax-H264Y-PAH) for *in vivo* activity determination. Hydrodynamic tail vein injection of these vectors showed that the PAH H264Y vector had similar activity to wild-type PAH in reducing serum Phe levels in adult PKU mice (Figure S2A). Consistent with this result, a bioinformatic analysis revealed that H264 is not conserved among different aromatic amino acid hydroxylases.

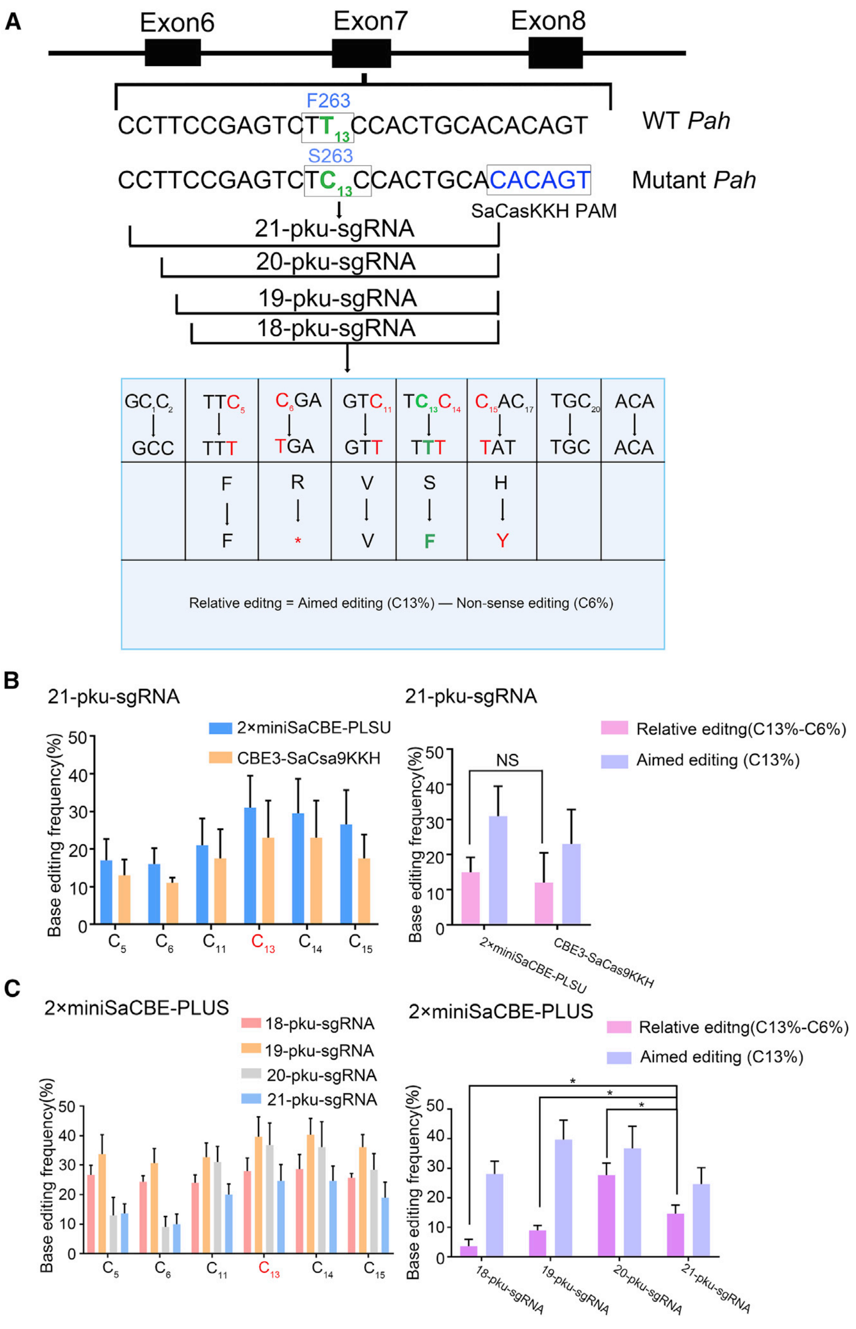

Figure 3. *In vitro* base editing of mouse PKU site by 2×miniSaCBE-PLUS

(A) Design of sgRNAs targeting *Pah*^{enu2} site. The spacer of a previous published 21-nt sgRNA was 5′ truncated to 20 nt, 19 nt, and 18 nt, respectively, all of which share a common PAM as indicated in blue. Lower table showed conversion of the amino acid codon by the base editing of each C. Bystander Cs are shown in red, and target C is shown in green. Conversion of target C (C13) leads to the desired correction (S263F) that restores PAH activity. Conversion of the bystander Cs leads to synonymous (C5, C11, and C14), non-sense (C6, R260*), and missense (C15, H 264 Y). H264Y mutation does not obviously impair the PAH activity, as shown in Figure S2A. (B) Editing of 2×miniSaCBE-PLUS and CBE3-SaCas9KKH with the 21-nt sgRNA on PKU site (n = 3 independent experiments). Values represent mean ± SD. Asterisks indicate statistically significant differences in functional editing efficiencies observed between CBE3-SaCas9KKH and 2×miniSaCBE-PLUS at PKU site. (C) Comparison of the editing efficiency of 5′ truncated PKU-sgRNAs coupled with 2×miniSaCBE-PLUS (n = 3 independent experiments). Values represent mean ± SD. Asterisks indicate statistically significant differences in functional editing efficiencies observed between different PKU-sgRNAs.

These hydroxylases, including phenylalanine, tryptophan, and tyrosine dehydrogenases, likely originated from a common ancestor and share a similar mechanism of action (Figure S2B).[20,21] Taken together, these analyses suggest that PAH H264Y is functional. Therefore, editing outcomes with C13T, but not C6T, would restore PAH function.

As shown in Figure 3B, the 21-nt PKU sgRNA induced a considerable amount of non-sense editing in both 2×miniSaCBE-PLUS and CBE3-SaCas9KKH edited cells. The relative editing efficiencies of C13 to C6 (C13%-C6%) were 15% in 2×miniSaCBE-PLUS and 12% in CBE3-SaCas9KKH cells, respectively. Next, we sought to optimize the editing strategy for minimizing the ratio of non-sense editing. Previous studies have observed that the length of the sgRNA spacer has an effect on the editing pattern of SpCas9-derived CBEs. To test whether we could minimize the frequency of non-sense editing by varying the length of sgRNAs, we constructed three additional PKU sgRNAs with truncated spacers ranging from 18 nt to 20 nt. As

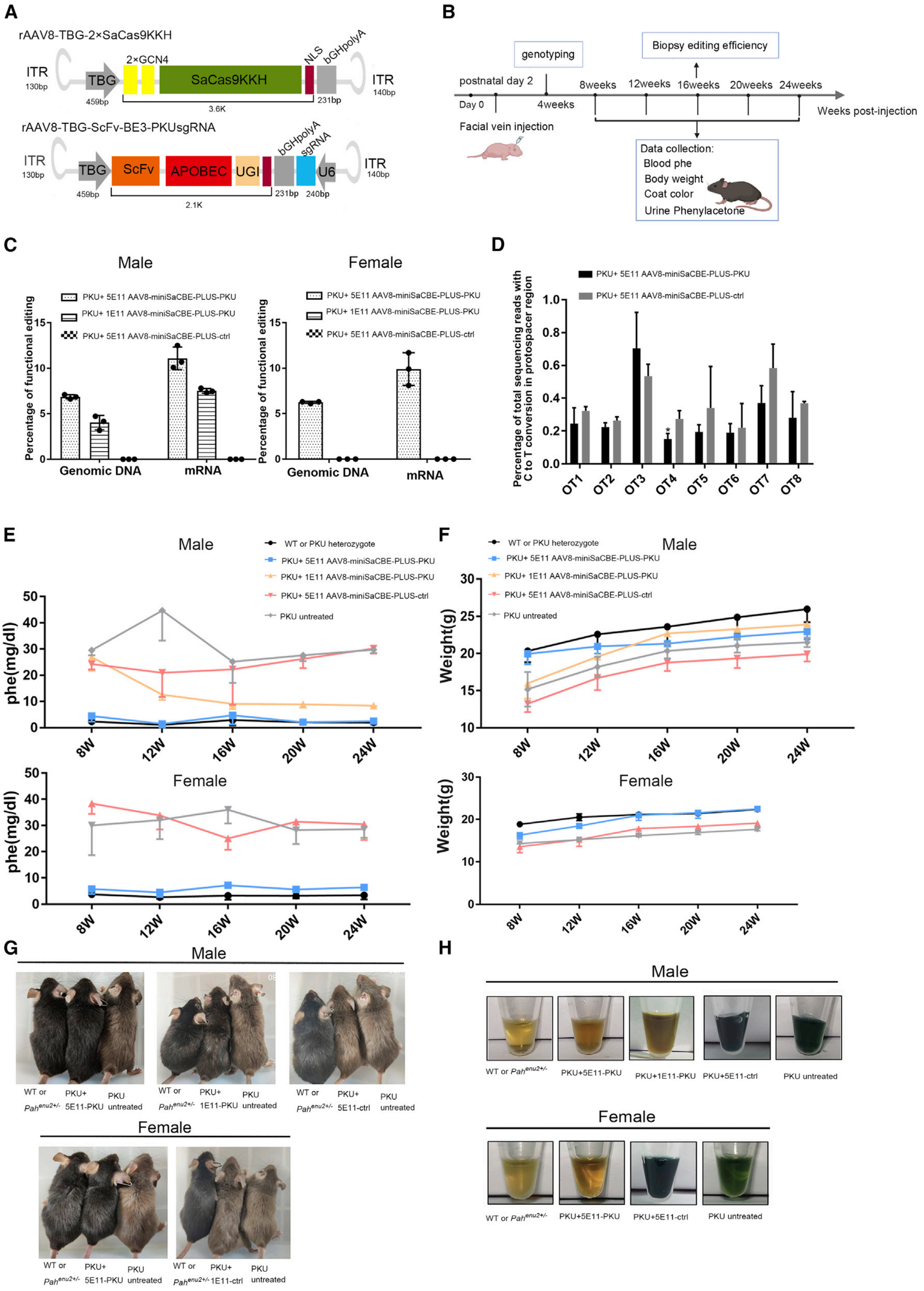

(legend on next page)

shown in Figure 3C, we observed that varying the spacer length affected the editing outcomes. The 20-nt spacer sgRNA significantly improved C13%-C6%, raising it to 27.7%. In addition, we also edited PKU sites using 1×minixCBE-PLUS and found that 1×minixCBE-PLUS was much less efficient than 1×minixCBE-PLUS (with 20nt-sgRNA) in terms of C13%-C6% (10% versus 27.7%) (Figures S3A and S3B).

Correction of PKU mutations by *in vivo* base editing

Based on the optimized PKU base editing strategy with our 2×miniSaCBE-PLUS system, we constructed all elements of the system as a pair of AAV vectors, with one expressing 2×GCN4-SaCas9KKH and the other expressing scFv-APOBEC1 and PKU-20 sgRNA or control sgRNA (Figure 4A). We chose AAV serotype 8 capsid because it can efficiently infect hepatocytes and reduce off-target infections.[22] We used a hepatocyte-specific human thyroxine-binding globulin (TBG) promoter to drive the expression of protein components of the 2×miniSaCBE-PLUS system to further restrict the action of base editing to hepatocytes. The experimental design of the gene therapy is depicted in Figure 4B. The AAV expressing the base editor with a non-targeting sgRNA served as a negative control (AAV8-miniSaCBE-PLUS-ctrl), and the AAV expressing the *Pah*-targeting base editor was the experimental group (AAV8-miniSaCBE-PLUS-PKU). AAVs were injected into 2-day-old newborn mice via facial vein at a dose of 1×10^{11} genome copies (GC)/per mouse (low-dose) or 5×10^{11} GC/per mouse (high-dose). For each dosage, we injected ~40 neonatal mice to obtain enough animals (>3) of both sexes. Mice were genotyped 4 weeks after infusion of AAVs. Data were collected from 8 to 24 weeks at 2-week intervals.

At 16 weeks after AAV infusion, genomic DNA and mRNA were extracted from liver biopsy specimens and subjected to polymerase chain reaction (PCR) amplification. High-throughput sequencing (HTS) was performed to quantify the editing of PKU mutations. As shown in Figure 4C, we did observe corrections for PKU mutations induced by AAV8-miniSaCBE-PLUS-PKU infusion. At the genomic DNA level, the average correction rates (C13T conversion) achieved 6.12%, 6.36%, and 3.58% in high-dose male mice, high-dose female mice, and low-dose male mice, respectively. Notably, the rates of non-sense editing (C6T) were minimal in all groups, with the highest being 0.29% (high-dose males) (Figure S4). In contrast, at the mRNA level, the average correction rates reached 9.81%, 10.24%, and 6.56% in high-dose male mice, high-dose female mice, and low-dose male mice, respectively. As *Pah* DNAs exist in all liver cells, while their mRNAs are expressed only in hepatocytes, the higher editing efficiency at the mRNA level further confirms the specificity of our system for hepatocytes. No obvious correction was observed in mice that received control AAV expressing a non-targeting sgRNA (Figure 4C).

After establishing that AAV8-miniSaCBE-PLUS-PKU infusion produced corrections for PKU mutations, we next tested whether off-target editing occurred. Genomic DNAs from mice injected with high doses of AAV8-miniSaCBE-PLUS-ctrl or AAV8-miniSaCBE-PLUS-PKU were extracted and subjected to PCR amplification from a set of eight off-target sites that were previously predicted based on their sequence similarity to PKU sgRNA(16). As shown in Figure 4D, we found no significant off-target editing by AAV8-miniSaCBE-PLUS-PKU.

Correction of phenylalanine metabolism by *in vivo* base editing

To examine whether correction of the PKU mutation improves phenylalanine metabolism and thus relieves hyperphenylalaninemia-related syndromes, a series of clinically relevant biochemical and behavioral tests were performed. Survey of blood phenylalanine levels showed that AAV8-miniSaCBE-PLUS-PKU infusion significantly rescued phenylalanine metabolism (Figure 4E). High doses of AAV-miniSaCBE-PLUS-PKU resulted in near-normalization of blood phenylalanine levels in both male mice (WT or PKU heterozygote versus PKU + 5×10^{11} AAV8-miniSaCBE-PLUS-PKU versus PKU + 5×10^{11} AAV8-miniSaCBE-PLUS-ctrl versus PKU untreated = 2.1 versus 2.1 versus 26.2 versus 27.6 mg/dL) and female mice (WT or PKU heterozygote versus PKU + 5×10^{11} AAV8-miniSaCBE-PLUS-PKU versus PKU + 5×10^{11} AAV8-miniSaCBE-PLUS-ctrl versus PKU untreated = 3.4 versus 6.4 versus 30.5 versus 28.6 mg/dL). Although the low dosage of AAV8-miniSaCBE-PLUS-PKU did not normalize blood phenylalanine, it did significantly reduce the level in PKU mice to about 32.2% (8.9 versus 27.6 mg/dL) (Figures 4E

Figure 4. *In vivo* base editing of *Pah*^{enu2} mouse corrected the *Pah*(F263S) mutation and normalized Phe metabolism

(A) Schematic maps showing the expression cassettes in AAV8 vector genomes. Hepatocyte-specific human TBG promoter was used to drive the expression of protein components of the base editor to ensure the base editing was limited to hepatocyte. (B) Time line of the *in vivo* experiments. Postnatal day 2 (P2) mice were fused with AAV8-miniSaCBE-PLUS-PKU or AAV8-miniSaCBE-PLUS-ctrl with dosage indicated in (C) (n = 3 mice for each group). Genotyping, sample collection, and analysis were performed at indicated time points. (C) *In vivo* editing efficiency of PKU target site in liver genomic DNA and mRNA determined by HTS (n = 3 mice for each group). Values represent mean ± SD. Asterisks indicate statistically significant differences in editing efficiencies observed between high-dose AAV8-miniSaCBE-PLUS-PKU-injected mice and low-dose AAV8-miniSaCBE-PLUS-PKU-injected mice. (D) Editing of 8 predicted off-target sites (OTs) in liver genomic DNA. OTs were predicted according to their sequence similarity to PKU target site[16] (n = 3 mice for each group). Values represent mean ± SD. Asterisks indicate statistically significant differences in editing efficiencies observed between high-dose AAV8-miniSaCBE-PLUS-PKU-injected mice and AAV8-miniSaCBE-PLUS-ctrl-injected mice. (E) Blood Phe levels from male (top) and female (bottom) PKU mice were determined every 2 weeks from 8 to 24 weeks (n = 3 mice for each group). Values represent mean ± SD. (F) Body weight growth charts of male (top) and female (bottom) PKU mice. Compared with untreated or AAV8-miniSaCBE-PLUS-ctrl-treated PKU mice, AAV8-miniSaCBE-PLUS-PKU-treated mice showed slightly relieved growth retardation (n = 3 mice for each group). Values represent mean ± SD. (G) AAV8-miniSaCBE-PLUS-PKU treatment rescue hypopigmented phenotype. High-dose (5×10^{11} GC) AAV8-miniSaCBE-PLUS-PKU (PKU+5×10^{11}) completely restored normal pigmentation in both male (3 of 3, n = 3) and female (3 of 3, n = 3) PKU mice. Low-dose AAV8-miniSaCBE-PLUS-PKU (PKU+1×10^{11}) partially rescued hypopigmentation in male PKU mice (3 of 3, n = 3). (H) AAV8-miniSaCBE-PLUS-PKU treatment reduced urinary Phe metabolites in male (top) and female (bottom) PKU mice.

and S5). Consistent with the improvement in blood phenylalanine levels, AAV8-miniSaCBE-PLUS-PKU treatment obviously rescued the growth retardation in PKU mice (Figures 4F and S5). Besides, the almost complete reversal of hypopigmentation in AAV8-miniSaCBE-PLUS-PKU mice made them indistinguishable from wild-type and heterozygous littermates, suggesting that melanin synthesis has been restored (Figure 4G). In contrast, AAV8-miniSaCBE-PLUS-ctrl-treated mice showed obvious growth retardation and hypopigmentation (Figure 4G). In addition, urine phenyl ketone was also significantly reduced by AAV8-miniSaCBE-PLUS-PKU treatment (Figure 4H). Accompanying the correction of phenylalanine metabolism, AAV8-miniSaCBE-PLUS-PKU treatment also improved the behavior of PKU mice. Compared with PKU mice that received AAV8-miniSaCBE-PLUS-ctrl or no treatment, AAV8-miniSaCBE-PLUS-PKU mice restored nesting and water maze behaviors to a large extent (Figure S6). In summary, these data demonstrate that *in vivo* base editing of AAV8-miniSaCBE-PLUS-PKU can correct phenylalanine metabolism and hyperphenylalaninemia-related syndromes.

DISCUSSION

Since the discovery of AAVs, much progress has been made in recent decades in the treatment of genetic diseases, especially liver-related metabolic disorders.[16,23,24] Compared with other viral vectors, rAAVs are relatively safe because they are less immunogenic and rarely integrate into the host genome.[15] With AAVs, particularly AAV8, showing tropism for hepatocytes,[22] many liver-related genetic disorders have been demonstrated to be curable in animal models.[25,26] Importantly, a clinical study showed that AAV8 vectors expressing FIX could be successfully delivered to the liver and clinically benefit patients with hemophilia B, demonstrating the translational potential of AAV8 vectors.[27] However, most of these AAV-based therapeutic strategies have been performed in adult animals rather than in neonates, possibly owing to the loss of the non-integrating AAV genome as developing hepatocytes proliferate.[28,29] In fact, in PKU patients, treatment at an early stage is extremely important to prevent secondary harmful effects in their developing organs, especially in the central nervous system.[12]

Genome editing technology, especially base editing tools, offers the opportunity to correct genetic lesions and thus cure these disorders at the start of life. Given that many genetic diseases, such as Gaucher disease (GD) and PKU, cause fatal death or irreversible damage to developing organs, early treatment of such diseases is extremely important. We showed that injection of AAVs encoding base editing tools into 2-day-old neonatal PKU mice corrected disease-causing mutations, resulting in normalization of Phe metabolism and correction of hyperphenylalaninemia (HPA)-related phenotypes. Surprisingly, PKU mice treated with base editing were undistinguishable from their healthy littermates in terms of appearance and behavior. By comparison, untreated PKU mice were much smaller in size and had lighter hairs than their littermates, which allowed them to be clearly distinguished. A previous study developed dual AAV base editors with an intein protein splicing system and achieved therapeutic correction of the adult PKU mouse model.[16] Therefore, our results, together with previous observations,[16] support the notion that genetic disorders caused by point mutations can be cured at both infant and adult stages by base editing technology. In addition, Cas9-mediated homologous direct repair (HDR) strategy was also used to treat neonatal PKU mouse models. In the HDR strategy, dual AAV vectors encoding Cas9 and repair templates, respectively, were used to repair the *Pah* mutation.[30] However, this strategy only partially rescued the phenylalanine disorder.[30]

Following base editing, the targeted mutation was corrected in about 36% of the alleles *in vitro*. A considerable portion of these corrections were accompanied by undesirable bystander editing, one of which generated a premature stop codon. This undoubtedly reduces the therapeutic effect. Therefore, narrowing the editing window to fit the target position should increase the ratio of correct editing and thus reduce the requirement for the number of AAVs. In addition to changing the sgRNA length, there are several ways to narrow the editing window. One is to mutate cytosine deaminase to reduce its catalytic activity. For example, base editors coupled to mutant APOBEC1 (W90Y, R126E) generally have a much narrower editing window than those coupled to wild-type APOBEC1.[31] Restricting the connection between deaminase and Cas9 by changing the flexible linker to an inflexible one is also helpful.[32]

As current base editors were developed from SpCas9 or SaCas9, their sizes (~5 kb for spCas9 base editors and ~4.2 kb for SaCas9 base editors) are larger than or very close to the packaging capacity of AAVs (~4.5 kb). Therefore, delivery of base editors via AAV is challenging. The protein splicing system from *Nostoc punctiforme* Npu intein has been introduced into base editors to split them into two expression cassettes, thereby fitting the packaging capacity of AAV.[16,33] Our procedure based on the minimal GCN4/scFv system also does not require splitting of Cas9 proteins. Importantly, considering the structural similarity between Cas9s from different organisms, our procedure will be generally applicable to other Cas9-derived base editors, such as cjCas9[34] and sauriCas9 etc.[35] Nevertheless, further research needs to be done to determine if the AAV-infected cells are removed by preexisting Cas9-specific immune responses, since the most widely used Cas9s, saCas9 and spCas9, are derived from common pathogens.[36–39] In addition, long-term investigations of AAV mediated base editing therapy are also required to evaluate the potential side effect of base editors, such as genome wide DNA and RNA off-target editing.[40–43]

MATERIALS AND METHODS

Plasmid construction

pCMV-2×GCN4-SaCas9KKH, pCMV-4×GCN4-SaCas9KKH, pCMV-ABE-scFv, pVax-H264Y-PAH, and pVax-WT-PAH were constructed through seamless cloning method (ClonExpress II One Step Cloning Kit; Vazyme Biotech). DNA sequences of these

constructed plasmids are listed in Note S1. For the construction of sgRNA plasmids, the corresponding spacers were inserted into each sgRNA expression plasmid digested with Bbs1. Spacer sequences for each sgRNAs are listed in Table S1. All plasmids were verified by Sanger sequencing.

Cell culture

HEK 293T cells were cultured in Dulbecco's modified Eagle's medium (Thermo Fisher Scientific, Waltham, MA), supplemented with 10% (v/v) fetal bovine serum (Life Technologies), 1% penicillin/streptomycin (Boster Biological Technology) and maintained at 37°C with 5% CO_2.

Plasmids transfection

HEK 293T cells were seeded on 96-well plates (Biofil). Cells at a confluence of ~70%–80% were transfected with TransEasy^{TM} (Foregene). Briefly, 300 ng of base editor (BE) (150 ng GCN4-Cas9, 150 ng scFv-CBE/ABE) and 100 ng of sgRNA expression plasmids were transfected using 0.7 μL of Transeasy per well according to the manufacturer's instruction. At 72 h after transfection, genomic DNA from each well was extracted by 30 μL freshly prepared lysis buffer. The mixture was incubated at 55°C for 20 min and then was heat inactivated at 95°C for another 10 min. The resulting DNA lysate was subjected to PCR amplification and subsequent analysis.

Base editing analysis with Sanger sequencing and EditR software

On-target genomic regions of interest were amplified by PCR and were analyzed with Sanger sequencing. Then, the sequencing graphs were further quantified by EditR software (baseditr.com).[44] Primers used for amplifying each target loci are listed in Table S2.

PAH activity evaluation

Adult PKU mice were used for evaluating the activity of wild-type and mutant PAH (H264Y). The plasmids encoding wild-type (pVax-WT-PAH) or mutant PAH (pVax-H264Y-PAH) were injected into PKU mice through hydrodynamic tail vein injection at a dosage of 4 mg/kg. Control PKU mice were injected with an equal volume of physiological saline. Blood samples were collected from the orbital vein at two time points, before the injection and 20–24 h after the injection. The samples were stored in the form of dried blood spots at 4°C. Phe concentration was determined with fluorescence spectrophotometry Phe detection kit (AN302). PAH activity was assessed by comparing the level of blood Phe before and after injection.

AAV8 vector production

AAV8-2×miniSaCBE-PLUS base editing system include two AAV vectors, one expressing 2×GCN4-SaCas9KKH (rAAV8-TBG-2×GCN4-SaCas9KKH) and the other one expressing scFv-APOBEC1-UGI and 20-PKU sgRNA (rAAV8-TBG-scFv-BE3-PKUsgRNA) or expressing scFv-APOBEC1-UGI (rAAV8-TBG-scFv-BE3-ctrl). The expression of both 2×SaCas9KKH and scFv-APOBEC1-UGI-PKU is driven by the hepatocyte-specific promoter (TBG). The expression of sgRNA is driven by the U6 promoter. To achieve specific liver targeting, the AAV plasmids were packaged into serotype 8 recombinant AAVs through a triple-transfection protocol as previously described.[45] All vectors used in AAV production passed the endotoxin assay using the endpoint chromogenic endotoxin test kit (Xiamen Bioendo Technology Xiamen, China). The presence of AAV2 inverted terminal repeats (ITRs) in the *cis*-acting AAV plasmids was examined by Sma1 digestion. The genome titer (GC/mL) of AAV8 vector was determined by quantitative PCR (qPCR) using forward primer 5′-GCCAGCCATCTG TTGT-3′, reverse primer 5′-GGAGTGGCACCTTCCA-3′, and probe 5′-Fam- TCCCCCGTGCCTTCCTTGACC-Tamra-3′. The purity of the resulting AAV8 vectors were determined by SDS-PAGE followed by SYPRO Ruby (Thermo Fisher Scientific) staining.

Animal care

The B6.BTBR-*Pah*^{enu2} mouse purchased from Jackson Laboratory carries a missense mutation (c.835T→C) in exon 7 (p.F263S) of the *Pah* gene (19). Developing homozygous *Pah*$^{enu2-/-}$ and *Pah*$^{enu2+/+}$ (WT) mice were issued from heterozygous mating *Pah*$^{enu2+/-}$. Animals were housed in specific-pathogen-free (SPF) animal breeding rooms and maintained on a 12-h light/dark cycle. All mice were provided with standard laboratory chow and water. All animal procedures were performed following the protocol approved by the Institutional Animal Care and Treatment Committee of Sichuan University (Chengdu, China) (IACUC number: 20,100,318).

Treatment of PKU

Two-day-old neonatal mice born of heterozygous PKU parents were used for AAV treatment. AAVs expressing rAAV8-TBG-2×GCN4-SaCas9KKH and rAAV8-TBG-scFv-BE3-PKUsgRNA or rAAV8-TBG-scFv-BE3-ctrlwere systematically injected through buccal vein, and each virus of the pair was injected at equal molar ratio in a volume of 50 μL (high dosage: 5 × 10^{11} GC/mouse; low dosage: 1 × 10^{11} GC/mouse). Untreated age-matched mice served as the untreated control group. At 4 weeks after AAV injection, mice were genotyped by examining DNAs from tail biopsies. Blood Phe, body weight, coat color, and urine phenylacetone were examined every 2 weeks from 8 to 24 weeks after AAV injection. Editing efficiency of the liver DNA and mRNA were determined at 16 weeks after injection.

Amplification and high-throughput DNA sequencing of genomic DNA and mRNA samples

Liver samples were collected by biopsy and subjected to genomic DNA and mRNA extraction. Genomic regions of interest were amplified by PCR with primers flanked with different barcodes (Table S3). cDNA was generated using All-in-One First-Strand cDNA Synthesis SuperMix (Innovagene) according to the manufacturer's protocol. Subsequent PCR reactions to generate amplicons for HTS were performed using 2×Rapid Taq Master Mix (Vazyme). The PCR product was purified using GeneJET Gel Extraction Kit (Thermo Fisher Scientific). Samples were sequenced commercially using Illumina Novaseq 6000 platform (Personalbio, Shanghai).

Primers used for amplifying each target loci are listed in Tables S3, S4, and S5.

High-throughput sequencing and data analysis

The sequencing reads were extracted from the .fastq file of deep sequencing and placed in a Microsoft EXCEL file. The upstream 30 bp and downstream 30 bp sequences of the spacer were removed. The search item was used to find the sequence edited by the base editors.

Blood Phe detection

According to previous reports,[46] the blood of mice was collected at 4 p.m. every day, and the whole blood was collected on the blood collection card, dried under ambient conditions, and stored at 4°C. The concentration of Phe in the blood was determined with a fluorescence spectrophotometry Phe detection kit (AN302) according to manufacturer's instructions.

Urinary phenylacetone detection

The ferric chloride test was used for the detection of phenylacetone in urine. Urine from each mouse was collected with a metabolism cage. Then, 30 μL urine was mixed with an equal volume of ferric chloride solution (10%). If phenylacetone was present in the sample, it formed a complex with the ferric chloride and developed an easily visualized deep green color.

Water maze assay

The ability of PKU mice to navigate to a visible platform was evaluated using the water maze and EthoVision video tracking software as previously described.[47] Five sessions were performed, with each session consisting of two trials. Each trial was up to 60 s with a 10- to 15-s interval. Time to reach the platform (latency), number of platform crossings, and swim speeds were calculated to assess the spatial navigational skill and memory of PKU mice.

Nest building assay

Nest building assay was performed as previously described with slight modification.[48] Briefly, each mouse was singly housed and provided with a cotton square to build a nest. Photos of the nests were taken 24 h after the mice were housed. Nest quality was scored according to a rating scale of 1 to 5 by two observers blinded to the treatment, where 1 indicates that the mouse has not torn the squares apart at all, and 5 indicates a highly complex nest. The scores from the two observers were averaged for statistical analysis.

Statistical analysis

GraphPad Prism v.8.4.0 software was used for all data analysis. Statistical comparison adjustment in Figures 1, 3, 4C, S1, S2, S3, and S5 was performed using two-tailed Student's *t*-test in IBM SPSS v.21.0 software. Statistical comparison adjustment in Figures 4E and 4F were performed using ANOVA in GraphPad Prism v.8.4.0 software. For all statistical comparisons, significance was noted as follows: *$p < 0.05$, **$p < 0.01$, ***$p < 0.001$.

ABBREVIATIONS

CRISPR-Cas Clustered regularly interspaced short palindromic repeats and CRISPR associated

sgRNAs Single-guide RNAs

AAV Adeno-associated virus

scFv Single chain variable fragment

PKU Phenylketonuria

DSBs Double-strand breaks

NHEJ Non-homologous end joining

HDR Homology-directed repair

NTS Non-target strand

TS Target strand

ssDNA Single-stranded DNA

PAH Phenylalanine hydroxylase

BE-PLUS programming larger C to U (T) scope

TadAs tRNA adenosine deaminases

Val/V Valine

His/H Histidine

Tyr/Y Tyrosine

Arg/R Arginine

HTS High-throughput sequencing

PCR Polymerase chain reaction

PAM Protospacer adjacent motif

HPA Hyperphenylalaninemia

UGI Uracil glycosylase inhibitor

GB1 G protein B1 domain

SUPPLEMENTAL INFORMATION

Supplemental information can be found online at https://doi.org/10.1016/j.omtm.2022.01.001.

ACKNOWLEDGMENTS

We thank Li Mo and Dengwei Gui for their help in AAV production and injection. We thank Dr. Heng Xu for help in deep-sequencing data analysis.

Funding: This work was supported by National Natural Science Foundation of China (No. U19A2002, No. 81771220 and No. 81974238).

AUTHOR CONTRIBUTIONS

L.Z., J.S., J.L, W.T., N.L, F.Q., Y.W., and Y.J. performed the experiments. Y.H., R.T., L.L., and L.J. analyzed the data. L.Z. and S.Y.

designed the experiments and wrote the manuscript. S.Y. and Y.Y. supervised the research.

DECLARATION OF INTERESTS

The authors declare no competing interests.

REFERENCES

1. Bolotin, A., Quinquis, B., Sorokin, A., and Ehrlich, S.D. (2005). Clustered regularly interspaced short palindrome repeats (CRISPRs) have spacers of extrachromosomal origin. Microbiology *151*, 2551–2561.
2. Mojica, F.J., Diez-Villasenor, C., Garcia-Martinez, J., and Soria, E. (2005). Intervening sequences of regularly spaced prokaryotic repeats derive from foreign genetic elements. J. Mol. Evol. *60*, 174–182.
3. Jinek, M., Chylinski, K., Fonfara, I., Hauer, M., Doudna, J.A., and Charpentier, E. (2012). A programmable dual-RNA-guided DNA endonuclease in adaptive bacterial immunity. Science *337*, 816–821.
4. Gasiunas, G., Barrangou, R., Horvath, P., and Siksnys, V. (2012). Cas9-crRNA ribonucleoprotein complex mediates specific DNA cleavage for adaptive immunity in bacteria. Proc. Natl. Acad. Sci. U S A *109*, E2579–E2586.
5. Perez-Pinera, P., Kocak, D.D., Vockley, C.M., Adler, A.F., Kabadi, A.M., Polstein, L.R., Thakore, P.I., Glass, K.A., Ousterout, D.G., Leong, K.W., et al. (2013). RNA-guided gene activation by CRISPR-Cas9-based transcription factors. Nat. Methods *10*, 973–976.
6. Thakore, P.I., D'Ippolito, A.M., Song, L., Safi, A., Shivakumar, N.K., Kabadi, A.M., Reddy, T.E., Crawford, G.E., and Gersbach, C.A. (2015). Highly specific epigenome editing by CRISPR-Cas9 repressors for silencing of distal regulatory elements. Nat. Methods *12*, 1143–1149.
7. Hilton, I.B., D'Ippolito, A.M., Vockley, C.M., Thakore, P.I., Crawford, G.E., Reddy, T.E., and Gersbach, C.A. (2015). Epigenome editing by a CRISPR-Cas9-based acetyltransferase activates genes from promoters and enhancers. Nat. Biotechnol. *33*, 510–517.
8. Anton, T., and Bultmann, S. (2017). Site-specific recruitment of epigenetic factors with a modular CRISPR/Cas system. Nucleus *8*, 279–286.
9. Komor, A.C., Kim, Y.B., Packer, M.S., Zuris, J.A., and Liu, D.R. (2016). Programmable editing of a target base in genomic DNA without double-stranded DNA cleavage. Nature *533*, 420–424.
10. Gaudelli, N.M., Komor, A.C., Rees, H.A., Packer, M.S., Badran, A.H., Bryson, D.I., and Liu, D.R. (2017). Programmable base editing of A*T to G*C in genomic DNA without DNA cleavage. Nature *551*, 464–471.
11. Nishimasu, H., Ran, F.A., Hsu, P.D., Konermann, S., Shehata, S.I., Dohmae, N., Ishitani, R., Zhang, F., and Nureki, O. (2014). Crystal structure of Cas9 in complex with guide RNA and target DNA. Cell *156*, 935–949.
12. Blau, N., van Spronsen, F.J., and Levy, H.L. (2010). Phenylketonuria. Lancet. *376*, 1417–1427.
13. Scriver, C.R., Eisensmith, R.C., Woo, S.L., and Kaufman, S. (1994). The hyperphenylalaninemias of man and mouse. Annu. Rev. Genet. *28*, 141–165.
14. Li, N., Jia, H., Liu, Z., Tao, J., Chen, S., Li, X., Deng, Y., Jin, X., Song, J., Zhang, L., et al. (2015). Molecular characterisation of phenylketonuria in a Chinese mainland population using next-generation sequencing. Sci. Rep. *5*, 15769.
15. Chen, Y.H., Keiser, M.S., and Davidson, B.L. (2018). Viral vectors for gene transfer. Curr. Protoc. Mouse Biol. *8*, e58.
16. Villiger, L., Grisch-Chan, H.M., Lindsay, H., Ringnalda, F., Pogliano, C.B., Allegri, G., Fingerhut, R., Haberle, J., Matos, J., Robinson, M.D., et al. (2018). Treatment of a metabolic liver disease by in vivo genome base editing in adult mice. Nat. Med. *24*, 1519–1525.
17. Jiang, W., Feng, S., Huang, S., Yu, W., Li, G., Yang, G., Liu, Y., Zhang, Y., Zhang, L., Hou, Y., et al. (2018). BE-PLUS: a new base editing tool with broadened editing window and enhanced fidelity. Cell Res. *28*, 855–861.
18. Tanenbaum, M.E., Gilbert, L.A., Qi, L.S., Weissman, J.S., and Vale, R.D. (2014). A protein-tagging system for signal amplification in gene expression and fluorescence imaging. Cell *159*, 635–646.
19. McDonald, J.D., Bode, V.C., Dove, W.F., and Shedlovsky, A. (1990). Pahhph-5: a mouse mutant deficient in phenylalanine hydroxylase. Proc. Natl. Acad. Sci. U S A *87*, 1965–1967.
20. Grenett, H.E., Ledley, F.D., Reed, L.L., and Woo, S.L. (1987). Full-length cDNA for rabbit tryptophan hydroxylase: functional domains and evolution of aromatic amino acid hydroxylases. Proc. Natl. Acad. Sci. U S A *84*, 5530–5534.
21. Ledley, F.D., DiLella, A.G., Kwok, S.C., and Woo, S.L. (1985). Homology between phenylalanine and tyrosine hydroxylases reveals common structural and functional domains. Biochemistry *24*, 3389–3394.
22. Gao, G.P., Alvira, M.R., Wang, L., Calcedo, R., Johnston, J., and Wilson, J.M. (2002). Novel adeno-associated viruses from rhesus monkeys as vectors for human gene therapy. Proc. Natl. Acad. Sci. U S A *99*, 11854–11859.
23. Yang, Y., Wang, L., Bell, P., McMenamin, D., He, Z., White, J., Yu, H., Xu, C., Morizono, H., Musunuru, K., et al. (2016). A dual AAV system enables the Cas9-mediated correction of a metabolic liver disease in newborn mice. Nat. Biotechnol. *34*, 334–338.
24. Rui, T.L.X. (2019). Long-term metabolic correction of phenylketonuria by AAV-delivered phenylalanine amino lyase. Methods Clin. Dev. *19*, 507–517.
25. Ding, Z., Georgiev, P., and Thony, B. (2006). Administration-route and gender-independent long-term therapeutic correction of phenylketonuria (PKU) in a mouse model by recombinant adeno-associated virus 8 pseudotyped vector-mediated gene transfer. Gene Ther. *13*, 587–593.
26. Miao, C.H., Nakai, H., Thompson, A.R., Storm, T.A., Chiu, W., Snyder, R.O., and Kay, M.A. (2000). Nonrandom transduction of recombinant adeno-associated virus vectors in mouse hepatocytes in vivo: cell cycling does not influence hepatocyte transduction. J. Virol. *74*, 3793–3803.
27. Nathwani, A.C., Tuddenham, E.G., Rangarajan, S., Rosales, C., McIntosh, J., Linch, D.C., Chowdary, P., Riddell, A., Pie, A.J., Harrington, C., et al. (2011). Adenovirus-associated virus vector-mediated gene transfer in hemophilia B. N. Engl. J. Med. *365*, 2357–2365.
28. Hu, C., Kasten, J., Park, H., Bhargava, R., Tai, D.S., Grody, W.W., Nguyen, Q.G., Hauschka, S.D., Cederbaum, S.D., and Lipshutz, G.S. (2014). Myocyte-mediated arginase expression controls hyperargininemia but not hyperammonemia in arginase-deficient mice. Mol. Ther. *22*, 1792–1802.
29. Wang, D., Tai, P.W.L., and Gao, G. (2019). Adeno-associated virus vector as a platform for gene therapy delivery. Nat. Rev. Drug Discov. *18*, 358–378.
30. Richards, D.Y., Winn, S.R., Dudley, S., Nygaard, S., Mighell, T.L., Grompe, M., and Harding, C.O. (2020). AAV-mediated CRISPR/Cas9 gene editing in murine phenylketonuria. Mol. Ther. Methods Clin. Dev. *17*, 234–245.
31. Kim, Y.B., Komor, A.C., Levy, J.M., Packer, M.S., Zhao, K.T., and Liu, D.R. (2017). Increasing the genome-targeting scope and precision of base editing with engineered Cas9-cytidine deaminase fusions. Nat. Biotechnol. *35*, 371–376.
32. Tan, J., Zhang, F., Karcher, D., and Bock, R. (2019). Author Correction: engineering of high-precision base editors for site-specific single nucleotide replacement. Nat. Commun. *10*, 2019.
33. Lim, C.K.W., Gapinske, M., Brooks, A.K., Woods, W.S., Powell, J.E., Zeballos, C.M., Winter, J., Perez-Pinera, P., and Gaj, T. (2020). Treatment of a mouse model of ALS by in vivo base editing. Mol. Ther. *28*, 1177–1189.
34. Yamada, M., Watanabe, Y., Gootenberg, J.S., Hirano, H., Ran, F.A., Nakane, T., Ishitani, R., Zhang, F., Nishimasu, H., and Nureki, O. (2017). Crystal structure of the minimal Cas9 from Campylobacter jejuni reveals the molecular diversity in the CRISPR-Cas9 systems. Mol. Cell *65*, 1109–1121 e3.
35. Hu, Z., Wang, S., Zhang, C., Gao, N., Li, M., Wang, D., Wang, D., Liu, D., Liu, H., Ong, S.G., et al. (2020). A compact Cas9 ortholog from Staphylococcus Auricularis (SauriCas9) expands the DNA targeting scope. PLoS Biol. *18*, e3000686.
36. Charlesworth, C.T., Deshpande, P.S., Dever, D.P., Camarena, J., Lemgart, V.T., Cromer, M.K., Vakulskas, C.A., Collingwood, M.A., Zhang, L., Bode, N.M., et al. (2019). Identification of preexisting adaptive immunity to Cas9 proteins in humans. Nat. Med. *25*, 249–254.
37. Wang, D., Mou, H., Li, S., Li, Y., Hough, S., Tran, K., Li, J., Yin, H., Anderson, D.G., Sontheimer, E.J., et al. (2015). Adenovirus-mediated somatic genome editing of Pten

by CRISPR/Cas9 in mouse liver in spite of Cas9-specific immune responses. Hum. Gene Ther. *26*, 432–442.

38. Hakim, C.H., Kumar, S.R.P., Perez-Lopez, D.O., Wasala, N.B., Zhang, D., Yue, Y., Teixeira, J., Pan, X., Zhang, K., Million, E.D., et al. (2021). Cas9-specific immune responses compromise local and systemic AAV CRISPR therapy in multiple dystrophic canine models. Nat. Commun. *12*, 6769.

39. Li, A., Tanner, M.R., Lee, C.M., Hurley, A.E., De Giorgi, M., Jarrett, K.E., Davis, T.H., Doerfler, A.M., Bao, G., Beeton, C., et al. (2020). AAV-CRISPR gene editing is negated by Pre-existing immunity to Cas9. Mol. Ther. *28*, 1432–1441.

40. Jin, S., Zong, Y., Gao, Q., Zhu, Z., Wang, Y., Qin, P., Liang, C., Wang, D., Qiu, J.L., Zhang, F., et al. (2019). Cytosine, but not adenine, base editors induce genome-wide off-target mutations in rice. Science *364*, 292–295.

41. Grunewald, J., Zhou, R., Garcia, S.P., Iyer, S., Lareau, C.A., Aryee, M.J., and Joung, J.K. (2019). Transcriptome-wide off-target RNA editing induced by CRISPR-guided DNA base editors. Nature *569*, 433–437.

42. Zuo, E., Sun, Y., Wei, W., Yuan, T., Ying, W., Sun, H., Yuan, L., Steinmetz, L.M., Li, Y., and Yang, H. (2019). Cytosine base editor generates substantial off-target single-nucleotide variants in mouse embryos. Science *364*, 289–292.

43. Zhou, C., Sun, Y., Yan, R., Liu, Y., Zuo, E., Gu, C., Han, L., Wei, Y., Hu, X., Zeng, R., et al. (2019). Off-target RNA mutation induced by DNA base editing and its elimination by mutagenesis. Nature *571*, 275–278.

44. Kluesner, M.G., Nedveck, D.A., Lahr, W.S., Garbe, J.R., Abrahante, J.E., Webber, B.R., and Moriarity, B.S. (2018). EditR: a method to quantify base editing from sanger sequencing. CRISPR J. *1*, 239–250.

45. Li, Q., Su, J., Liu, Y., Jin, X., Zhong, X., Mo, L., Wang, Q., Deng, H., and Yang, Y. (2021). In vivo PCSK9 gene editing using an all-in-one self-cleavage AAV-CRISPR system. Mol. Ther. Methods Clin. Dev. *20*, 652–659.

46. Zeile, W.L., McCune, H.C., Musson, D.G., O'Donnell, B., O'Neill, C.A., Tsuruda, L.S., Zori, R.T., and Laipis, P.J. (2018). Maternal phenylketonuria syndrome: studies in mice suggest a potential approach to a continuing problem. Pediatr. Res. *83*, 889–896.

47. Winn, S.R., Scherer, T., Thony, B., Ying, M., Martinez, A., Weber, S., Raber, J., and Harding, C.O. (2018). Blood phenylalanine reduction corrects CNS dopamine and serotonin deficiencies and partially improves behavioral performance in adult phenylketonuric mice. Mol. Genet. Metab. *123*, 6–20.

48. Johnson, L.A., Zuloaga, D.G., Bidiman, E., Marzulla, T., Weber, S., Wahbeh, H., and Raber, J. (2015). ApoE2 exaggerates PTSD-related behavioral, cognitive, and neuroendocrine alterations. Neuropsychopharmacology *40*, 2443–2453.

OMTM, Volume *24*

Supplemental information

A universal strategy for AAV delivery of base editors to correct genetic point mutations in neonatal PKU mice

Lifang Zhou, Jing Su, Jie Long, Rui Tao, Wenling Tang, Fengming Qin, Nan Liu, Yanhong Wang, Yaoge Jiao, Yun Hu, Lurong Jiang, Li Li, Yang Yang, and Shaohua Yao

Supplementary information

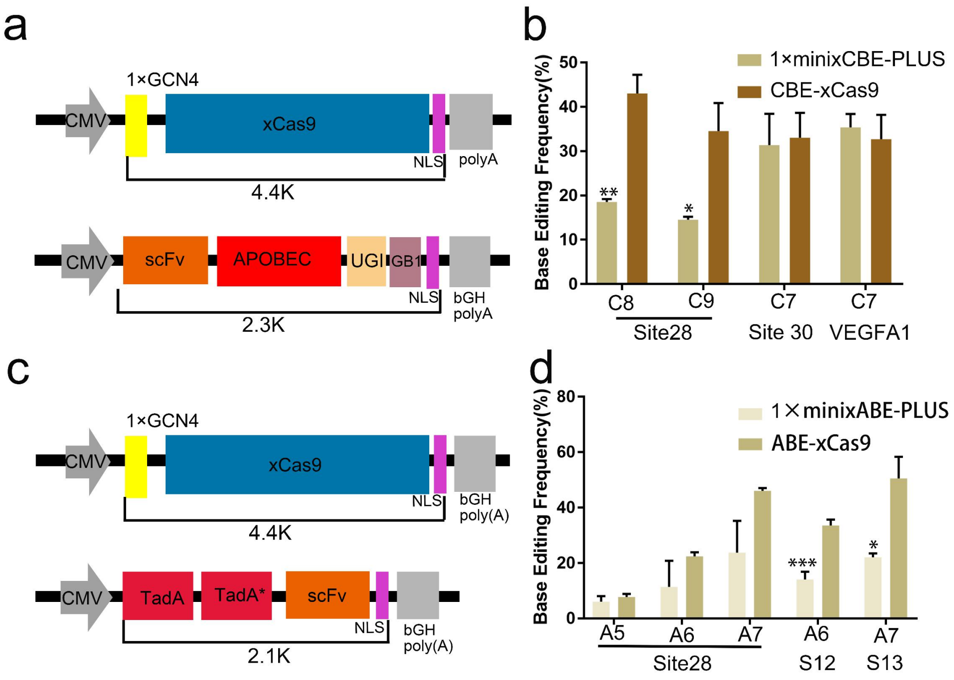

Figure S1. Construction and verification of minixBE-PLUS editing system. (a) Schematic diagram showing the expression cassettes of pCMV-1×GCN4-xCas9, CMV-scFv-CBE. (b) Cytosine base editing of 1×minixCBE-PLUS and CBE-xCas9 at three genomic loci in HEK293T cells. (n = 3 independent experiments). Values represent mean ± SD. Asterisks indicate statistically significant differences in editing efficiencies observed between CBE-xCas9 and 1×minixCBE-PLUS at each site. (c) Schematic diagram showing the expression cassettes of pCMV-1×GCN4-xCas9, pCMV-ABE-scFv. (d) Adenine base editing of 1×minixABE-PLUS and ABE-xCas9 at three genomic loci in HEK293T cells. (n = 3 independent experiments). Values represent mean ± SD. Asterisks indicate statistically significant differences in editing efficiencies observed between ABE-xCas9 and 1×minixABE-PLUS at each site. (abbreviations, CMV(human cytomegalovirus promoter), UGI(Uracil DNA

glycosylase inhibitor), NLS (nuclear localization signal), GB1(G protein B1 domain))

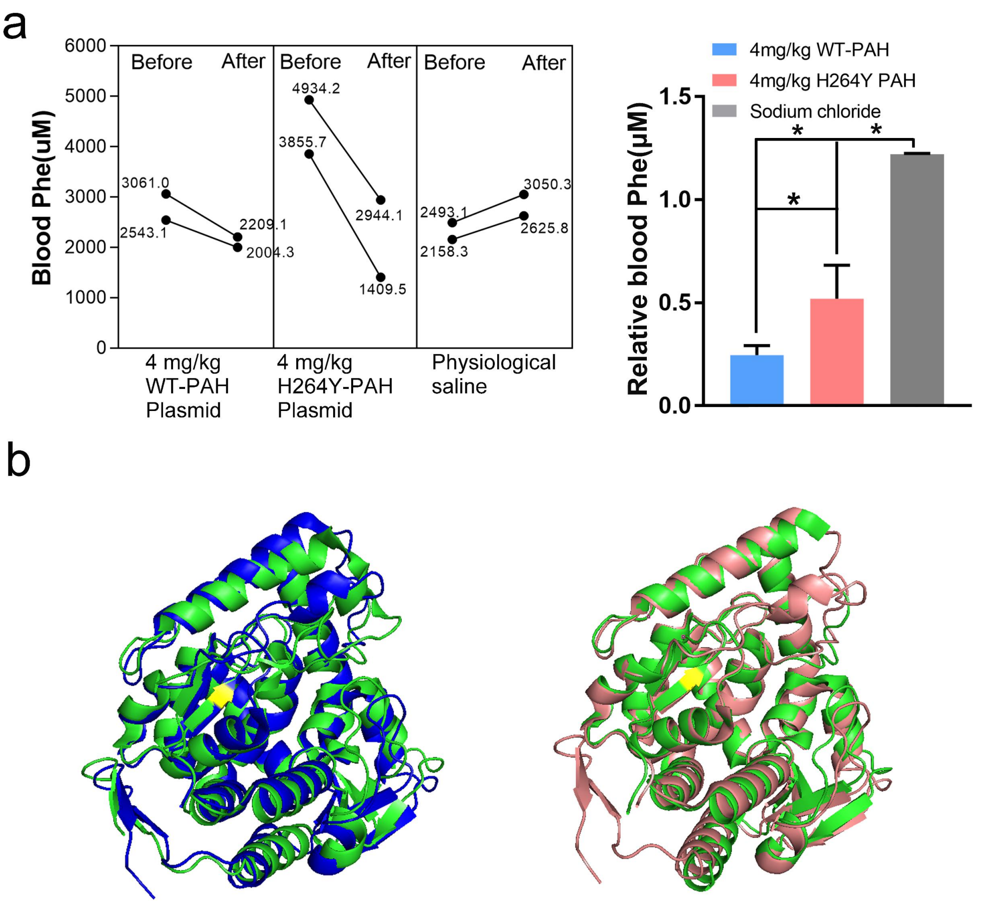

Figure S2. PAH activity evaluation. (a) H264Y-Pah enzyme activity verification. Changes of blood Phe in mice after plasmid injection (left panel). The relative changes of Phe in mice blood after plasmid injection (right panel) (n = 2 or 3 independent experiments). Values represent mean ± SD. Asterisks indicate statistically significant differences in functional editing efficiencies observed between WT-PAH or H254Y-PAH plasmid injected mice and sodium chloride injected mice. (b) Comparison of protein structure between phenylalanine hydroxylase monomer (PDB, 6hpo) (green) and tyrosine hydroxylase monomer (blue) (PDB, 3hfb), or

tryptophan hydroxylase monomer(pink) (PDB, 13e2t). position F264 of phenylalanine hydroxylase is marked in yellow.

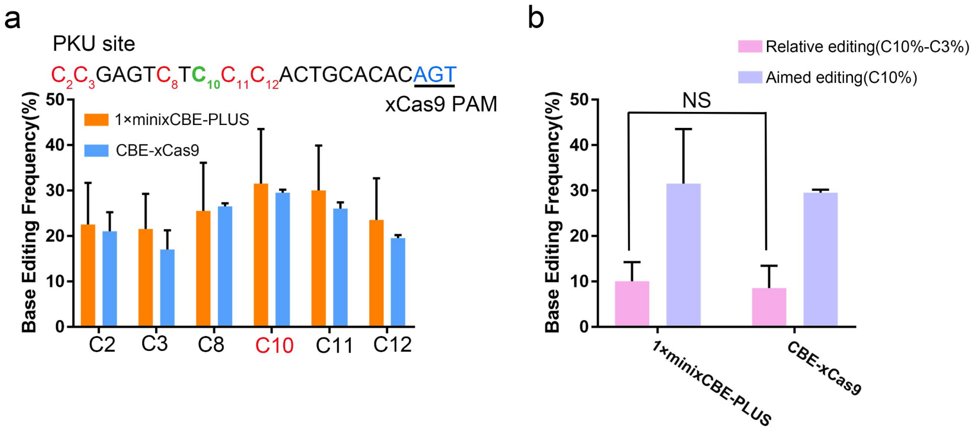

Figure S3. ***In vitro*** **base editing of mouse PKU site by 1×minixCBE-PLUS.** (a) The editing of 1×minixCBE-PLUS, and CBE-xCas9 with the sgRNA on PKU site of each c (n = 3 independent experiments). The aimed cytosine (C10, i.e. C835) was shown in green, and the bystanders were shown in red. (b) The relative editing efficiency (C10%-C3%) of 1×minixCBE-PLUS, and CBE-xCas9 (n = 3 independent experiments). Values represent mean ± SD. Asterisks indicate statistically significant differences in functional editing efficiencies observed between CBE-xCas9 and 1×minixCBE-PLUS at PKU site .

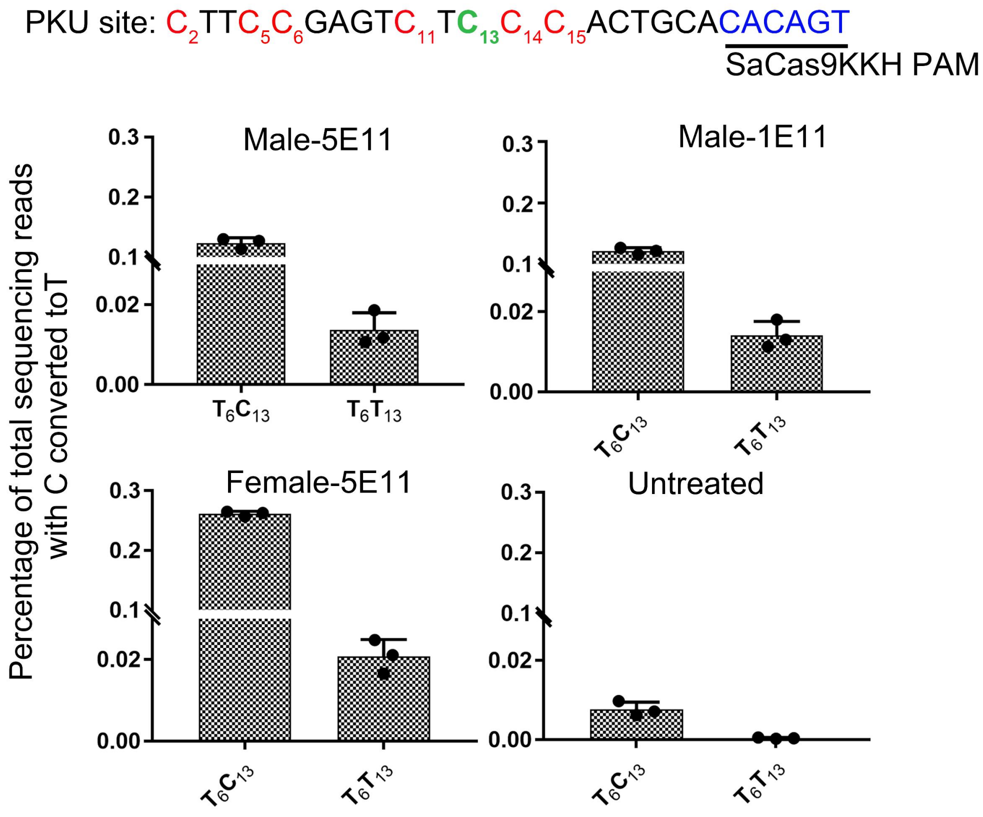

Figure S4. AAV8-miniSaCBE-PLUS-PKU produced minus non-sense edits *in vivo.* HTS of high-dose (5E11-PKU) male, low-dose (1E11-PKU) male, high-dose (5E11-PKU) female, untreated mice liver DNA revealed that AAV8-miniSaCBE-PLUS-PKU only produced minus non-sense edits *in vivo.*

Groups \ Weeks	Phe										Weigh									
	Male					Female					Male					Female				
	8w	12w	16w	20w	24w	8w	12w	16w	20w	24w	8w	12w	16w	20w	24w	8w	12w	16w	20w	24w
WT or PKU heterozygote vs 5E11 AAV8-miniSaCBE-PLUS-PKU	ns	ns	ns	ns	ns	ns	ns	ns	ns	ns	ns	ns	ns	ns	ns	**	*	ns	ns	ns
WT or PKU heterozygote vs 1E11 AAV8-miniSaCBE-PLUS-PKU	****	*	ns	ns	ns						**	ns	ns	ns	ns					
WT or PKU heterozygote vs 5E11 AAV8-miniSaCBE-PLUS-ctrl	****	****	****	****	****	****	****	****	****	****	****	****	**	***	****	****	****	***	**	***
WT or PKU heterozygote vs PKU untreated	****	****	****	****	****	****	****	****	****	****	***	**	ns	*	**	****	****	****	****	****
5E11 AAV8-miniSaCBE-PLUS-PKU vs 1E11 AAV8-miniSaCBE-PLUS-PKU	****	*	ns	ns	ns						*	ns	ns	ns	ns					
5E11 AAV8-miniSaCBE-PLUS-PKU vs 5E11 AAV8-miniSaCBE-PLUS-ctrl	****	****	****	****	****	****	****	****	****	****	****	**	ns	ns	ns	**	***	***	***	***
5E11 AAV8-miniSaCBE-PLUS-PKU vs PKU untreated	****	****	****	****	****	****	****	****	****	****	**	ns	ns	ns	ns	ns	**	****	****	****
1E11 AAV8-miniSaCBE-PLUS-PKU vs 5E11 AAV8-miniSaCBE-PLUS-ctrl	ns	ns	**	****	****						ns	ns	*	*	*					
1E11 AAV8-miniSaCBE-PLUS-PKU vs PKU untreated	ns	****	***	****	****						ns	ns	ns	ns	ns					
5E11 AAV8-miniSaCBE-PLUS-ctrl vs PKU untreated	ns	****	ns	ns	ns	ns	ns	*	ns	ns	ns	ns	ns	ns	ns	ns	ns	ns	ns	ns

Figure S5. The results of *ANOVA* analysis between the mutiple groups in

Figure 4e and Figure 4f. Asterisks indicate statistically significant differences in phe level and weigh of tested mice between multiple groups.

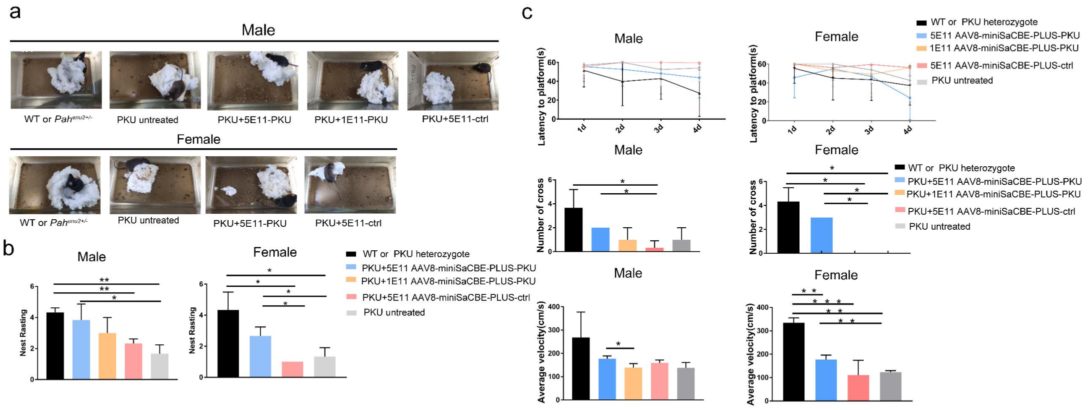

Figure S6. Behavioral analysis. (a) Photos of male and female mice' nesting situation. (b) Rating assessment of mice's nesting ability. n=3 mice for each group. Values represent mean ± SD. Asterisks indicate statistically significant differences between each group. (c) Performance in the water maze is presented with male and female respectively. The results are shown in top latency to the target platform (seconds), middle number of crosses, bottom average velocity (cm/s). n=3 mice for each group. Values represent mean ± SD. Asterisks indicate statistically significant differences between each group.

Table S1. List of sgRNAs and Oligos sequence

sgRNA	target sequence	Oligo F	Oligo R
FANCF site1	GATGTTCCAATCAGTACGCA **GAGAGT**	CACCGATGTTCCAATCAGTACGCA	AAACTGCGTACTGATTGGAACATC
CYP4V2 site4	TGCCTTAGATATCATCTGTG **GAGAAT**	CACCGTGCCTTAGATATCATCTGTG	AAACCACAGATGATATCTAAGGCAC
HEK3-1	TCTGCTTCTCCAGCCCTGGC **CTGGGT**	CACCGTCTGCTTCTCCAGCCCTGGC	AAACGCCAGGGCTGGAGAAGCAGAC
HEK293 site4-4	CTTTAACCCCCACCTCCAGC **CGCAGT**	CACCGCTTTAACCCCCACCTCCAGC	AAACGCTGGAGGTGGGGGTTAAAGC
EMX1	CCTCCCTCCCTGGCCCAGGT **GAAGGT**	CACCGCCTCCCTCCCTGGCCCAGGT	AAACACCTGGGCCAGGGAGGGAGGC
FANCF site2	GCCGTCTCCAAGGTGAAAGC **GGAAGT**	CACCGCCGTCTCCAAGGTGAAAGC	AAACGCTTTCACCTTGGAGACGGC
Sa site6	GATGTTCCAATCAGTACGCA **GAGAGT**	CACCGATGTTCCAATCAGTACGCA	AAACTGCGTACTGATTGGAACATCC
VEGFA site11	GCTCCATTCACCCAGCTTCCC **TGTGGT**	CACCGCTCCATTCACCCAGCTTCCC	AAACGGGAAGCTGGGTGAATGGAGC
RUNX1 4	GTACTCACCTCTCATGAAGCACT **GTGGGT**	CACCGTACTCACCTCTCATGAAGCACT	AAACAGTGCTTCATGAGAGGTGAGTAC
EMX1 #6	GCAACCACAAACCCACGAGGG **CAGAGT**	CACCGCAACCACAAACCCACGAGGG	AAACCCCTCGTGGGTTTGTGGTTGC
Sa site1	CTGAATAGCTGCAAACAAGTG **CAGAAT**	CACCGCTGAATAGCTGCAAACAAGTG	AAACCACTTGTTTGCAGCTATTCAGC
Sa site11	GCTGTTGCATGAGGAAAGGGAC **TAGAGT**	CACCGCTGTTGCATGAGGAAAGGGAC	AAACGTCCCTTTCCTCATGCAACAGC
Site28	GACAAACCAGAAGCCGCTCC **TGG**	CACCGACAAACCAGAAGCCGCTCC	AAACGGAGCGGCTTCTGGTTTGTCC
Site30	GAACACAAAGCATAGACTGC **GGG**	CACCGAACACAAAGCATAGACTGC	AAACGCAGTCTATGCTTTGTGTTCC

VEGFA 1	GATGTCTGCAG GCCAGATGA **GGG**	CACCGATGTCTG CAGGCCAGATGA	AAACTCATCTGGCCT GCAGACATC
S12	GCAGACGGCAG TCACTAGGG **GGC**	CACCGCAGACGG CAGTCACTAGGG	AAACCCCTAGTGACT GCCGTCTGC
S13	GTCGCAGGACA GCTTTTCCT **AGA**	CACCGTCGCAGG ACAGCTTTTCCT	AAACAGGAAAAGCTG TCCTGCGAC
18-PKU-sgRNA	TCCGAGTCTCC CACTGCA **CACAGT**	CACCGTCCGAGT CTTCCACTGCA	AAACTGCAGGGGAAG ACTGGAC
19-PKU-sgRNA	TTCCGAGTCTCC CACTGCA **CACAGT**	CACCGTTCCGAG TCTTCCACTGCA	AAACTGCAGGGGAAG ACTGGAAC
20-PKU-sgRNA	CTTCCGAGTCTC CCACTGCA **CACAGT**	CACCGCTTCCGA GTCTTCCACTGC A	AAACTGCAGGGGAAG ACTGGAAGC
21-PKU-sgRNA	CCTTCCGAGTCT TCCACTGCA **CACAGT**	CACCGCCTTCCG AGTCTTCCACTG CA	AAACTGCAGTGGAAG ACTGGAAGGC

Table S2. Primers used to amplify each target regions for sanger sequencing

Target site	chrom	Forward primer	Reverse primer
FANCF site1	Chr11	CGACGAGACAAAGGCG GCT	TCACTGTGACGTCCTGCT CT
CYP4V2 site4	Chr4	TGAAGAAACTTCGGTAT CTG	TATGGAAGCCCATGCAG GCG
HEK3-1	Chr9	AGAATGGGTCACAGTG GCAA	TAGGAAAAGCTGTCCTG CGA
HEK293 site4-4	Chr20	CTGCTGAGGGCGGCTTC TC	TGAAATCGCTCGGAGCC TC
EMX1	Chr2	TCTCTCTGGCCCACTGT GTC	CCATTGGCCTGCTTCGTG
FANCF site2	Chr11	CGACGAGACAAAGGCG GCT	TCACTGTGACGTCCTGCT CT
Sa site6	Chr11	ATGACTGGCATCATCTC GCA	GGTGCTGACGTAGGTAG TGC
VEGFA11	Chr6	GGAACAAGGGCCTCTGT CTG	GCCGTTCCCTCTTTGCTA GG
RUNX14	Chr2	GTTCTCACGCACCGACT	GAGTCCCAGAGGTATCC

	1	GAA	AGC
EMX1 #6	Chr2	TCTCTCTGGCCCACTGT GTC	CCATTGGCCTGCTTCGTG
Sa site1	Chr5	AAGTTACTGCAGCCCAA G	CAAGCAGGTGATTACAG G
Sa site11	Chr1	GCAGAAACCACAGTGT GT	CACTACCCCTGTTCCTAA AG
Site28	Chr3	GGCACAAAGGATGAAG GCT	GCTCAGTCTTGCATGAA ACAC
Site30	Chr5	ACAGGCTACCCCCTAAG T	TCCCAAGTGAGAAGCCA GTG
VEGFA site1	Chr6	GGAACAAGGGCCTCTGT CTG	GCCGTTCCCTCTTTGCTA GG
S12	Chr6	CACAGCTTCCCGTTCTC AGC	AGGGACACACAGATCTA TT
S13	Chr9	ATGTGGGCTGCCTAGAA AGG	CCCAGCCAAACTTGTCA ACC
PKU-sgRNA		CCTTGGGGAGTCATACC TCA	ATAAAGCAGGCAGTGGA TCA
BGH_PA_qP CR		GCCAGCCATCTGTTGT	GGAGTGGCACCTTCCA
BGH_PA_qP CR_Probe		TCCCCCGTGCCTTCCTT GACC	

Table S3. HTS Primers used for on-target sequence amplification.

Primer name	Forward primer	Reverse primer
1-Bar-DNA-OT	aagcgtg CCTTGGGGAGTCATACCTCA	GTTCAGGTG
2-Bar-DNA-OT	attctcg CCTTGGGGAGTCATACCTCA	TGTACATGG
3-Bar-DNA-OT	attgctc CCTTGGGGAGTCATACCTCA	
4-Bar-DNA-OT	atcaagc CCTTGGGGAGTCATACCTCA	
5-Bar-DNA-OT	atccgga CCTTGGGGAGTCATACCTCA	
6-Bar-DNA-OT	atcgaag CCTTGGGGAGTCATACCTCA A	
7-Bar-DNA-OT	atcgtcc CCTTGGGGAGTCATACCTCA	
8-Bar-DNA-OT	atgcttc CCTTGGGGAGTCATACCTCA	
9-Bar-DNA-OT	atggcga CCTTGGGGAGTCATACCTCA	
10-Bar-DNA-OT	acttagc CCTTGGGGAGTCATACCTCA	
S99-Bar-DNA-OT	tgtgtca CCTTGGGGAGTCATACCTCA	
S38-Bar-DNA-OT	caactgt CCTTGGGGAGTCATACCTCA	
1-RNA-pku-ontargetF	catcgac GACAACATCCCGCAGCT	GTTCAGGTG
2-RNA-pku-ontargetF	catgatc GACAACATCCCGCAGCT	TGTACATGG
3-RNA-pku-ontargetF	catgcaa GACAACATCCCGCAGCT	
4-RNA-pku-ontargetF	cagttac GACAACATCCCGCAGCT	

5-RNA-pku-ontargetF	ctatggt GACAACATCCCGCAGCT
6-RNA-pku-ontargetF	ctacaag GACAACATCCCGCAGCT
7-RNA-pku-ontargetF	ctacgtc GACAACATCCCGCAGCT
8-RNA-pku-ontargetF	ctagcac GACAACATCCCGCAGCT
9-RNA-pku-ontargetF	cttagct GACAACATCCCGCAGCT
10-RNA-pku-ontargetF	cttggag GACAACATCCCGCAGCT
11-RNA-pku-ontargetF	ctgctaa GACAACATCCCGCAGCT
12-RNA-pku-ontargetF	ctcgtat GACAACATCCCGCAGCT
13-RNA-pku-ontargetF	gaactac GACAACATCCGCAGCT
14-RNA-pku-ontargetF	gaaccta GACAACATCCCGCAGCT
15-RNA-pku-ontargetF	gaaggtc GACAACATCCCGCAGCT
16-RNA-pku-ontargetF	gattctg GACAACATCCCGCAGCT
17-RNA-pku-ontargetF	gattgct GACAACATCCCGCAGCT
18-RNA-pku-ontargetF	gatgacg GACAACATCCCGCAGCT
19-RNA-pku-ontargetF	gatgtgc GACAACATCCCGCAGCT
20-RNA-pku-ontargetF	gaccatc GACAACATCCCGCAGCT
21-RNA-pku-ontargetF	gacgaat GACAACATCCCGCAGCT

Table S4. Off-target sequence

Target site	sequence	chrom	strand
OT1	ACTTCAGACTCTCTCACTGCAACTGGT	Chr14	-
OT2	ATTTCCAAGTCTCCCACTGCTGTGAGT	Chr10	+
OT3	CCTCCCCAGTCTCCCACTGCTGACAAT	Chr11	-
OT4	CCTACAGAGTATTCCACTGCACCCAGT	Chr10	-
OT5	CCTTTCGAGTCTTTCACTGCACTCAGT	Chr7	-
OT6	TCTTCCTAGGCTCCCACAGCAATGAGT	Chr11	-
OT7	CATTCCCAGTGTACCACTGCAGTCAGT	Chr4	+
OT8	TCTTCAGAGTCTTCCTCTGCATCCGAT	ChrX	+

Table S5. Off-target Primers for amplification

off-target site	Forward primer	Reverse primer
OT1	gctgtaa GCATTTCTCAGATCTAATCTTCTGA	TGAGCATATGTGACCT GAGGA
OT2	gcctcta GAAACAGTTTGCACCCCCTA	CCCCAGGTTCATCAGT TCAC
OT3	gcgagtt CTTAGCGCCGCTGACTCT	TCCCTCTCCTTTTGCTT CCT
OT4	aactagg GACTGCAATACTCACGGTTCC	TGAGCCCAAGAGACTT CCTG
OT5	aacctag GCCATTTGATGAATGAATAGCA	GGTTTTCCATCCGTCC TGT

OT6	aagatgc AGGATGAGACCCAGGACCA	CCTGCAAGCTTTCCAC TCAT
OT7	ctggatg GCTGGGGCTATACAAAATTCC	CAGTAGTTCCAGTCAC GGTTG
OT8	ccaatga AAAAATATGCCCGTGTCATTG	TGCTGCTGTTGGAATC TGAG

Note1:

Plasmid sequences (CDS)

4×GCN4-SaCas9KKH

ATGGAGGAACTGCTGAGCAAGAACTACCACCTGGAAAACGAGGTGGCCA
GACTGAAGAAAGGCTCTGGCTCTGGCGGAAGCGGTTCTGGCGGATCAGGA
TCTGGAAGTGGCGGCTCTGGAAGCGGAGGTTCTGGATCTGGCGAAGAACT
GCTGTCTAAGAATTATCACCTCGAGAACGAAGTGGCTCGGCTCAAGAAAG
GCAGTGGCAGCGGAGGAAGTGGATCCGGCGGTAGTGGTAGTGGAAGCGG
CGGATCCGGCTCAGGCGGATCTGGTTCAGGGGAAGAACTCCTCTCCAAAA
ACTACCATCTCGAGAATGAGGTCGCCCGCCTGAAAAAAGGATCAGGTTCC
GGTGGTTCTGGCAGCGGTGGTTCAGGCTCAGGCAGCGGCGGTAGCGGTAG
CGGTGGAAGCGGAAGCGGAGAAGAACTTCTCAGCAAAAATTACCACTTG
GAGAATGAAGTTGCAAGACTCAAAAAAGGTTCCGGCAGTGGCGGCAGCG
GCAGCGGCGGATCTGGTAGTGGATCTGGTGGCAGTGGTTCAGGCGGAAGT
GGTAGCGGACCCGGGACCTCAGAGTCCGCCACACCCGAAAGTGGGAAAC
GGAACTACATCCTGGGGCTTGCCATTGGGATAACCAGCGTTGGCTACGGA
ATTATTGATTATGAGACACGCGATGTGATTGACGCCGGGGTTAGGCTGTTC
AAAGAGGCCAACGTTGAAAACAACGAGGGAAGACGGAGTAAGCGCGGA
GCAAGAAGACTCAAGCGCAGACGGAGACATCGGATTCAGAGGGTGAAAA
AGCTGCTCTTCGATTACAATCTCCTGACCGATCATAGTGAGCTGAGCGGA
ATCAACCCCTACGAGGCGCGAGTGAAAGGGCTTTCCCAGAAGCTGTCCGA
AGAGGAGTTCTCCGCCGCGTTGCTGCACCTGGCCAAACGGAGGGGGTTC
ACAATGTAAACGAAGTGGAGGAGGACACGGGCAATGAACTTAGTACGAA
AGAACAGATCAGTAGGAACTCTAAGGCTCTCGAAGAGAAATACGTCGCTG
AGTTGCAGCTTGAGAGACTGAAAAAAGACGGCGAAGTACGCGGATCTATT
AATAGGTTCAAGACTTCAGATTACGTAAAGGAAGCCAAGCAGCTCCTGAA
AGTACAGAAAGCGTACCATCAGCTCGATCAGAGCTTCATCGATACCTACA
TAGATTTGCTGGAGACACGGAGGACATACTACGAGGGCCCAGGGGAAGG
ATCTCCTTTTGGGTGGAAGGACATCAAGGAATGGTACGAGATGCTTATGG
GACATTGTACATATTTTCCGGAGGAGCTCAGGAGCGTCAAGTACGCCTAC
AATGCCGACCTGTACAATGCCCTCAATGACCTCAATAACCTCGTGATTACC
AGGGACGAGAACGAGAAGCTGGAGTACTATGAAAAGTTCCAGATTATCG
AGAATGTGTTTAAGCAGAAGAAGAAGCCGACACTTAAGCAGATTGCAAA
GGAAATCCTCGTGAATGAGGAAGATATCAAGGGATACAGAGTGACAAGT
ACAGGCAAGCCCGAGTTCACAAATCTGAAGGTGTACCACGATATTAAGGA
CATAACCGCACGAAAGGAGATAATCGAAAACGCTGAGCTCCTCGATCAG

ATCGCAAAAATTCTTACCATCTACCAGTCTAGTGAGGACATTCAGGAGGA
ACTGACTAATCTGAACAGTGAGCTCACCCAAGAGGAAATTGAGCAGATTT
CAAACCTGAAAGGCTACACCGGGACGCACAATCTGAGCCTCAAAGCAATC
AACCTCATTCTGGATGAACTTTGGCACACAAATGACAACCAAATTGCCAT
ATTCAACCGCCTGAAACTGGTGCCAAAAAAAGTGGATCTGTCACAGCAAA
AGGAAATCCCTACAACCTTGGTTGACGATTTTATTCTGTCCCCCGTTGTCA
AGCGGAGCTTCATCCAGTCAATCAAGGTGATCAATGCCATCATTAAAAAA
TACGGATTGCCAAACGATATAATTATCGAGCTTGCACGAGAGAAGAACTC
AAAGGACGCCCAGAAGATGATTAACGAAATGCAGAAGCGCAACCGCCAG
ACAAACGAACGCATAGAGGAAATTATAAGAACAACCGGCAAAGAGAATG
CCAAGTATCTGATCGAGAAAATCAAGCTGCACGACATGCAAGAAGGCAA
GTGCCTGTACTCTCTGGAAGCTATCCCACTCGAAGATCTGCTGAATAATCC
ATTCAATTACGAGGTGGACCACATCATCCCTAGATCCGTAAGCTTTGACA
ATTCCTTCAATAACAAAGTTCTGGTTAAACAGGAGGAAAATTCTAAAAAA
GGGAACCGGACCCCGTTCCAGTACCTGAGCTCCAGTGACAGCAAGATTAG
CTACGAGACTTTTAAGAAACATATTCTGAATCTGGCCAAAGGCAAAGGCA
GGATCAGCAAGACCAAGAAGGAGTACCTCCTCGAAGAACGCGACATTAA
CAGATTTAGTGTGCAGAAAGATTTCATCAACCGAAACCTTGTCGATACTC
GGTACGCCACGAGAGGCCTGATGAATCTCCTCAGGAGCTACTTCCGCGTC
AATAATCTGGACGTTAAAGTCAAGAGCATAAATGGGGGATTCACCAGCTT
TCTGAGGAGAAAGTGGAAGTTTAAGAAGGAACGAAACAAAGGATACAAG
CACCATGCTGAGGATGCTTTGATCATCGCTAACGCGGACTTTATCTTTAAG
GAATGGAAAAAGCTGGATAAGGCAAAGAAAGTGATGGAAAACCAGATGT
TCGAGGAGAAGCAGGCAGAGTCAATGCCTGAGATCGAGACAGAGCAGGA
ATACAAGGAAATTTTCATCACCCCTCATCAGATTAAACACATAAAGGACT
TCAAAGACTATAAATACTCTCATAGGGTGGACAAAAAACCCAATCGCAAG
CTCATTAATGACACCCTGTACTCAACACGGAAGGATGATAAAGGTAATAC
CTTGATTGTGAATAATCTTAATGGATTGTATGACAAAGATAACGACAAGC
TCAAGAAGCTGATCAACAAGTCTCCAGAGAAGCTCCTTATGTATCACCAC
GACCCACAGACTTATCAGAAATTGAAACTGATCATGGAGCAATACGGGGA
TGAGAAGAACCCACTCTACAAATATTATGAGGAAACAGGTAATTACCTGA
CCAAGTACTCCAAGAAGGATAACGGACCAGTGATCAAAAAGATAAAGTA
CTATGGCAACAAACTTAATGCGCATTTGGACATAACTGACGATTACCCCA
ATTCTCGAAACAAGGTTGTGAAGCTCTCCCTGAAGCCTTATAGATTTGACG
TGTACCTGGATAATGGGGTTTATAAATTCGTCACCGTGAAAAATCTGGAC
GTGATCAAAAAGGAGAACTATTATGAAGTAAACTCAAAGTGCTATGAGGA
GGCGAAGAAGCTGAAGAAGATCTCCAATCAGGCCGAGTTCATCGCTTCCT
TCTATAAGAACGATCTCATCAAGATCAATGGAGAGCTTTATCGCGTCATT
GGTGTGAACAATGACTTGCTGAACAGGATCGAAGTCAATATGATAGACAT
TACCTACCGGGAGTATCTCGAAAACATGAATGATAAACGGCCGCCTCACA
TCATCAAGACAATCGCATCTAAAACTCAGTCAATAAAAAAGTACTCTACC
GATATCCTGGGGAATCTCTATGAAGTGAAGTCAAAGAAGCACCCACAAAT
CATTAAAAAAGGTGGATCCCCCAAGAAGAAGAGGAAAGTCTCGAGCGAC
TACAAAGACCATGACGGTGATTATAAAGATCATGACATCGATTACAAGGA

TGACGATGACAAGTCTGGTGGTTCTACTAATCTGTCAGATATTATTGAAAA
GGAGACCGGTAAGCAACTGGTTATCCAGGAATCCATCCTCATGCTCCCAG
AGGAGGTGGAAGAAGTCATTGGGAACAAGCCGGAAAGCGATATACTCGT
GCACACCGCCTACGACGAGAGCACCGACGAGAATGTCATGCTTCTGACTA
GCGACGCCCCTGAATACAAGCCTTGGGCTCTGGTCATACAGGATAGCAAC
GGTGAGAACAAGATTAAGATGCTCTCTGGTGGTTCTCCCAAGAAGAAGAG
GAAAGTCTAA

2×GCN4-SaCas9KKH

ATGGAGGAACTGCTGAGCAAGAACTACCACCTGGAAAACGAGGTGGCCA
GACTGAAGAAAGGCTCTGGCTCTGGCGGAAGCGGTTCTGGCGGATCAGGA
TCTGGAAGTGGCGGCTCTGGAAGCGGAGGTTCTGGATCTGGCGAAGAACT
GCTGTCTAAGAATTATCACCTCGAGAACGAAGTGGCTCGGCTCAAGAAAG
GCAGTGGCAGCGGAGGAAGTGGATCCGGCGGTAGTGGTAGTGGAAGCGG
CGGATCCGGCTCAGGCGGATCTGGTTCAGGGCCCGGGACCTCAGAGTCCG
CCACACCCGAAAGTGGGAAACGGAACTACATCCTGGGGCTTGCCATTGGG
ATAACCAGCGTTGGCTACGGAATTATTGATTATGAGACACGCGATGTGAT
TGACGCCGGGGTTAGGCTGTTCAAAGAGGCCAACGTTGAAAACAACGAG
GGAAGACGGAGTAAGCGCGGAGCAAGAAGACTCAAGCGCAGACGGAGA
CATCGGATTCAGAGGGTGAAAAAGCTGCTCTTCGATTACAATCTCCTGAC
CGATCATAGTGAGCTGAGCGGAATCAACCCCTACGAGGCGCGAGTGAAA
GGGCTTTCCCAGAAGCTGTCCGAAGAGGAGTTCTCCGCCGCGTTGCTGCA
CCTGGCCAAACGGAGGGGGGTTCACAATGTAAACGAAGTGGAGGAGGAC
ACGGGCAATGAACTTAGTACGAAAGAACAGATCAGTAGGAACTCTAAGG
CTCTCGAAGAGAAATACGTCGCTGAGTTGCAGCTTGAGAGACTGAAAAAA
GACGGCGAAGTACGCGGATCTATTAATAGGTTCAAGACTTCAGATTACGT
AAAGGAAGCCAAGCAGCTCCTGAAAGTACAGAAAGCGTACCATCAGCTC
GATCAGAGCTTCATCGATACCTACATAGATTTGCTGGAGACACGGAGGAC
ATACTACGAGGGCCCAGGGGAAGGATCTCCTTTTGGGTGGAAGGACATCA
AGGAATGGTACGAGATGCTTATGGGACATTGTACATATTTTCCGGAGGAG
CTCAGGAGCGTCAAGTACGCCTACAATGCCGACCTGTACAATGCCCTCAA
TGACCTCAATAACCTCGTGATTACCAGGGACGAGAACGAGAAGCTGGAGT
ACTATGAAAAGTTCCAGATTATCGAGAATGTGTTTAAGCAGAAGAAGAAG
CCGACACTTAAGCAGATTGCAAAGGAAATCCTCGTGAATGAGGAAGATAT
CAAGGGATACAGAGTGACAAGTACAGGCAAGCCCGAGTTCACAAATCTG
AAGGTGTACCACGATATTAAGGACATAACCGCACGAAAGGAGATAATCG
AAAACGCTGAGCTCCTCGATCAGATCGCAAAAATTCTTACCATCTACCAG
TCTAGTGAGGACATTCAGGAGGAACTGACTAATCTGAACAGTGAGCTCAC
CCAAGAGGAAATTGAGCAGATTTCAAACCTGAAAGGCTACACCGGGACG
CACAATCTGAGCCTCAAAGCAATCAACCTCATTCTGGATGAACTTTGGCA
CACAAATGACAACCAAATTGCCATATTCAACCGCCTGAAACTGGTGCCAA
AAAAAGTGGATCTGTCACAGCAAAAGGAAATCCCTACAACCTTGGTTGAC
GATTTTATTCTGTCCCCCGTTGTCAAGCGGAGCTTCATCCAGTCAATCAAG
GTGATCAATGCCATCATTAAAAAATACGGATTGCCAAACGATATAATTAT

CGAGCTTGCACGAGAGAAGAACTCAAAGGACGCCCAGAAGATGATTAAC
GAAATGCAGAAGCGCAACCGCCAGACAAACGAACGCATAGAGGAAATTA
TAAGAACAACCGGCAAAGAGAATGCCAAGTATCTGATCGAGAAAATCAA
GCTGCACGACATGCAAGAAGGCAAGTGCCTGTACTCTCTGGAAGCTATCC
CACTCGAAGATCTGCTGAATAATCCATTCAATTACGAGGTGGACCACATC
ATCCCTAGATCCGTAAGCTTTGACAATTCCTTCAATAACAAAGTTCTGGTT
AAACAGGAGGAAAATTCTAAAAAAGGGAACCGGACCCCGTTCCAGTACC
TGAGCTCCAGTGACAGCAAGATTAGCTACGAGACTTTTAAGAAACATATT
CTGAATCTGGCCAAAGGCAAAGGCAGGATCAGCAAGACCAAGAAGGAGT
ACCTCCTCGAAGAACGCGACATTAACAGATTTAGTGTGCAGAAAGATTTC
ATCAACCGAAACCTTGTCGATACTCGGTACGCCACGAGAGGCCTGATGAA
TCTCCTCAGGAGCTACTTCCGCGTCAATAATCTGGACGTTAAAGTCAAGA
GCATAAATGGGGGATTCACCAGCTTTCTGAGGAGAAAGTGGAAGTTTAAG
AAGGAACGAAACAAAGGATACAAGCACCATGCTGAGGATGCTTTGATCA
TCGCTAACGCGGACTTTATCTTTAAGGAATGGAAAAAGCTGGATAAGGCA
AAGAAAGTGATGGAAAACCAGATGTTCGAGGAGAAGCAGGCAGAGTCAA
TGCCTGAGATCGAGACAGAGCAGGAATACAAGGAAATTTTCATCACCCCT
CATCAGATTAAACACATAAAGGACTTCAAAGACTATAAATACTCTCATAG
GGTGGACAAAAAACCCAATCGCAAGCTCATTAATGACACCCTGTACTCAA
CACGGAAGGATGATAAAGGTAATACCTTGATTGTGAATAATCTTAATGGA
TTGTATGACAAAGATAACGACAAGCTCAAGAAGCTGATCAACAAGTCTCC
AGAGAAGCTCCTTATGTATCACCACGACCCACAGACTTATCAGAAATTGA
AACTGATCATGGAGCAATACGGGGATGAGAAGAACCCACTCTACAAATAT
TATGAGGAAACAGGTAATTACCTGACCAAGTACTCCAAGAAGGATAACG
GACCAGTGATCAAAAAGATAAAGTACTATGGCAACAAACTTAATGCGCAT
TTGGACATAACTGACGATTACCCCAATTCTCGAAACAAGGTTGTGAAGCT
CTCCCTGAAGCCTTATAGATTTGACGTGTACCTGGATAATGGGGTTTATAA
ATTCGTCACCGTGAAAAATCTGGACGTGATCAAAAAGGAGAACTATTATG
AAGTAAACTCAAAGTGCTATGAGGAGGCGAAGAAGCTGAAGAAGATCTC
CAATCAGGCCGAGTTCATCGCTTCCTTCTATAAGAACGATCTCATCAAGAT
CAATGGAGAGCTTTATCGCGTCATTGGTGTGAACAATGACTTGCTGAACA
GGATCGAAGTCAATATGATAGACATTACCTACCGGGAGTATCTCGAAAAC
ATGAATGATAAACGGCCGCCTCACATCATCAAGACAATCGCATCTAAAAC
TCAGTCAATAAAAAAGTACTCTACCGATATCCTGGGGAATCTCTATGAAG
TGAAGTCAAAGAAGCACCCACAAATCATTAAAAAAGGTGGATCCCCCAA
GAAGAAGAGGAAAGTCTCGAGCGACTACAAAGACCATGACGGTGATTAT
AAAGATCATGACATCGATTACAAGGATGACGATGACAAGTCTGGTGGTTC
TACTAATCTGTCAGATATTATTGAAAAGGAGACCGGTAAGCAACTGGTTA
TCCAGGAATCCATCCTCATGCTCCCAGAGGAGGTGGAAGAAGTCATTGGG
AACAAGCCGGAAAGCGATATACTCGTGCACACCGCCTACGACGAGAGCA
CCGACGAGAATGTCATGCTTCTGACTAGCGACGCCCCTGAATACAAGCCT
TGGGCTCTGGTCATACAGGATAGCAACGGTGAGAACAAGATTAAGATGCT
CTCTGGTGGTTCTCCCAAGAAGAAGAGGAAAGTCTAA

1×GCN4-xCas9

ATGGGACCCAAGAAAAAACGCAAGGTGGAAGATCCTAAGAAAAAGCGG
AAAGTGGACGGCATTGGTAGTGGGAGCAACGGCAGCAGCGGAGAGGAAC
TGCTGAGCAAGAACTACCACCTGGAAAACGAGGTGGCCAGACTGAAGAA
AGGCTCTGGCTCTGGCGGAAGCGGTTCTGGCGGATCAGGATCTGGAAGTG
GCGGCGACAAGAAGTACTCCATTGGGCTCGCTATCGGCACAAACAGCGTC
GGCTGGGCCGTCATTACGGACGAGTACAAGGTGCCGAGCAAAAAATTCA
AAGTTCTGGGCAATACCGATCGCCACAGCATAAAGAAGAACCTCATTGGC
GCCCTCCTGTTCGACTCCGGGGAGACGGCCGAAGCCACGCGGCTCAAAAG
AACAGCACGGCGCAGATATACCCGCAGAAAGAATCGGATCTGCTACCTGC
AGGAGATCTTTAGTAATGAGATGGCTAAGGTGGATGACTCTTTCTTCCATA
GGCTGGAGGAGTCCTTTTTGGTGGAGGAGGATAAAAAGCACGAGCGCCA
CCCAATCTTTGGCAATATCGTGGACGAGGTGGCGTACCATGAAAAGTACC
CAACCATATATCATCTGAGGAAGAAGCTTGTAGACAGTACTGATAAGGCT
GACTTGCGGTTGATCTATCTCGCGCTGGCGCATATGATCAAATTTCGGGGA
CACTTCCTCATCGAGGGGGACCTGAACCCAGACAACAGCGATGTCGACAA
ACTCTTTATCCAACTGGTTCAGACTTACAATCAGCTTTTCGAAGAGAACCC
GATCAACGCATCCGGAGTTGACGCCAAAGCAATCCTGAGCGCTAGGCTGT
CCAAATCCCGGCGGCTCGAAAACCTCATCGCACAGCTCCCTGGGGAGAAG
AAGAACGGCCTGTTTGGTAATCTTATCGCCCTGTCCCTCGGGCTGACCCCC
AACTTTAAATCTAACTTCGACCTGGCCGAAGATACCAAGCTTCAACTGAG
CAAAGACACCTACGATGATGATCTCGACAATCTGCTGGCCCAGATCGGCG
ACCAGTACGCAGACCTTTTTTTGGCGGCAAAGAACCTGTCAGACGCCATT
CTGCTGAGTGATATTCTGCGAGTGAACACGGAGATCACCAAAGCTCCGCT
GAGCGCTAGTATGATCAAGCTCTATGATGAGCACCACCAAGACTTGACTT
TGCTGAAGGCCCTTGTCAGACAGCAACTGCCTGAGAAGTACAAGGAAATT
TTCTTCGATCAGTCTAAAAATGGCTACGCCGGATACATTGACGGCGGAGC
AAGCCAGGAGGAATTTTACAAATTTATTAAGCCCATCTTGGAAAAAATGG
ACGGCACCGAGGAGCTGCTGGTAAAGCTTAACAGAGAAGATCTGTTGCGC
AAACAGCGCACTTTCGACAATGGAATCATCCCCCACCAGATTCACCTGGG
CGAACTGCACGCTATCCTCAGGCGGCAAGAGGATTTCTACCCCTTTTTGAA
AGATAACAGGGAAAAGATTGAGAAAATCCTCACATTTCGGATACCCTACT
ATGTAGGCCCCCTCGCCCGGGGAAATTCCAGATTCGCGTGGATGACTCGC
AAATCAGAAGAGACCATCACTCCCTGGAACTTCGAGAAAGTCGTGGATAA
GGGGGCCTCTGCCCAGTCCTTCATCGAAAGGATGACTAACTTTGATAAAA
ATCTGCCTAACGAAAAGGTGCTTCCTAAACACTCTCTGCTGTACGAGTACT
TCACAGTTTATAACGAGCTCACCAAGGTCAAATACGTCACAGAAGGGATG
AGAAAGCCAGCATTCCTGTCTGGAGATCAGAAGAAAGCTATTGTGGACCT
CCTCTTCAAGACGAACCGGAAAGTTACCGTGAAACAGCTCAAAGAAGACT
ATTTCAAAAAGATTGAATGTTTCGACTCTGTTGAAATCAGCGGAGTGGAG
GATCGCTTCAACGCATCCCTGGGAACGTATCACGATCTCCTGAAAATCATT
AAAGACAAGGACTTCCTGGACAATGAGGAGAACGAGGACATTCTTGAGG
ACATTGTCCTCACCCTTACGTTGTTTGAAGATAGGGAGATGATTGAAGAA
CGCTTGAAAACTTACGCTCATCTCTTCGACGACAAAGTCATGAAGCAGCT

CAAGAGGCGCCGATATACAGGATGGGGGCGGCTGTCAAGAAAACTGATC
AATGGGATCCGAGACAAGCAGAGTGGAAAGACAATCCTGGATTTTCTTAA
GTCCGATGGATTTGCCAACCGGAACTTCATTCAGTTGATCCATGATGACTC
TCTCACCTTTAAGGAGGACATCCAGAAAGCACAAGTTTCTGGCCAGGGGG
ACAGTCTTCACGAGCACATCGCTAATCTTGCAGGTAGCCCAGCTATCAAA
AAGGGAATACTGCAGACCGTTAAGGTCGTGGATGAACTCGTCAAAGTAAT
GGGAAGGCATAAGCCCGAGAATATCGTTATCGAGATGGCCCGAGAGAAC
CAAACCACCCAGAAGGGACAGAAGAACAGTAGGGAAAGGATGAAGAGG
ATTGAAGAGGGTATAAAAGAACTGGGGTCCCAAATCCTTAAGGAACACCC
AGTTGAAAACACCCAGCTTCAGAATGAGAAGCTCTACCTGTACTACCTGC
AGAACGGCAGGGACATGTACGTGGATCAGGAACTGGACATCAATCGGCT
CTCCGACTACGACGTGGATCATATCGTGCCCCAGTCTTTTCTCAAAGATGA
TTCTATTGATAATAAAGTGTTGACAAGATCCGATAAAAACAGAGGGAAGA
GTGATAACGTCCCCTCAGAAGAAGTTGTCAAGAAAATGAAAAATTATTGG
CGGCAGCTGCTGAACGCCAAACTGATCACACAACGGAAGTTCGATAATCT
GACTAAGGCTGAACGAGGTGGCCTGTCTGAGTTGGATAAAGCCGGTTTCA
TCAAAAGGCAGCTTGTTGAGACACGCCAGATCACCAAGCACGTGGCCCAA
ATTCTCGATTCACGCATGAACACCAAGTACGATGAAAATGACAAACTGAT
TCGAGAGGTGAAAGTTATTACTCTGAAGTCTAAGCTGGTCTCAGATTTCAG
AAAGGACTTTCAGTTTTATAAGGTGAGAGAGATCAACAATTACCACCATG
CGCATGATGCCTACCTGAATGCAGTGGTAGGCACTGCACTTATCAAAAAA
TATCCCAAGCTTGAATCTGAATTTGTTTACGGAGACTATAAAGTGTACGAT
GTTAGGAAAATGATCGCAAAGTCTGAGCAGGAAATAGGCAAGGCCACCG
CTAAGTACTTCTTTTACAGCAATATTATGAATTTTTTCAAGACCGAGATTA
CACTGGCCAATGGAGAGATTCGGAAGCGACCACTTATCGAAACAAACGG
AGAAACAGGAGAAATCGTGTGGGACAAGGGTAGGGATTTCGCGACAGTC
CGGAAGGTCCTGTCCATGCCGCAGGTGAACATCGTTAAAAAGACCGAAGT
ACAGACCGGAGGCTTCTCCAAGGAAAGTATCCTCCCGAAAAGGAACAGC
GACAAGCTGATCGCACGCAAAAAAGATTGGGACCCCAAGAAATACGGCG
GATTCGATTCTCCTACAGTCGCTTACAGTGTACTGGTTGTGGCTAAAGTGG
AGAAAGGGAAGTCTAAAAAACTCAAAAGCGTCAAGGAACTGCTGGGCAT
CACAATCATGGAGCGATCAAGCTTCGAAAAAAACCCCATCGACTTTCTCG
AGGCGAAAGGATATAAAGAGGTCAAAAAAGACCTCATCATTAAGCTTCCC
AAGTACTCTCTCTTTGAGCTTGAAAACGGCCGGAAACGAATGCTCGCTAG
TGCGGGCGTGCTGCAGAAAGGTAACGAGCTGGCACTGCCCTCTAAATACG
TTAATTTCTTGTATCTGGCCAGCCACTATGAAAAGCTCAAAGGGTCTCCCG
AAGATAATGAGCAGAAGCAGCTGTTCGTGGAACAACACAAACACTACCTT
GATGAGATCATCGAGCAAATAAGCGAATTCTCCAAAAGAGTGATCCTCGC
CGACGCTAACCTCGATAAGGTGCTTTCTGCTTACAATAAGCACAGGGATA
AGCCCATCAGGGAGCAGGCAGAAAACATTATCCACTTGTTTACTCTGACC
AACTTGGGCGCGCCTGCAGCCTTCAAGTACTTCGACACTACCATAGACAG
AAAGCGGTACACCTCTACAAAGGAGGTCCTGGACGCCACACTGATTCATC
AGTCAATTACGGGGCTCTATGAAACAAGAATCGACCTCTCTCAGCTCGGT
GGAGACGAGGGAGCTGATAAGCGCACCGCCGATGGTTCCGAGTTCGAAA

GCCCCAAGAAGAAGAGGAAAGTCTAA

pCMV-ABE-scFv
ATGCACCAAAGAAGAAGCGGAAAGTCTCTGAAGTCGAGTTTAGCCACGA
GTATTGGATGAGGCACGCACTGACCCTGGCAAAGCGAGCATGGGATGAA
AGAGAAGTCCCCGTGGGCGCCGTGCTGGTGCACAACAATAGAGTGATCGG
AGAGGGATGGAACAGGCCAATCGGCCGCCACGACCCTACCGCACACGCA
GAGATCATGGCACTGAGGCAGGGAGGCCTGGTCATGCAGAATTACCGCCT
GATCGATGCCACCCTGTATGTGACACTGGAGCCATGCGTGATGTGCGCAG
GAGCAATGATCCACAGCAGGATCGGAAGAGTGGTGTTCGGAGCACGGGA
CGCCAAGACCGGCGCAGCAGGCTCCCTGATGGATGTGCTGCACCACCCCG
GCATGAACCACCGGGTGGAGATCACAGAGGGAATCCTGGCAGACGAGTG
CGCCGCCCTGCTGAGCGATTTCTTTAGAATGCGGAGACAGGAGATCAAGG
CCCAGAAGAAGGCACAGAGCTCCACCGACTCTGGAGGATCTAGCGGAGG
ATCCTCTGGAAGCGAGACACCAGGCACAAGCGAGTCCGCCACACCAGAG
AGCTCCGGCGGCTCCTCCGGAGGATCCTCTGAGGTGGAGTTTTCCCACGA
GTACTGGATGAGACATGCCCTGACCCTGGCCAAGAGGGCACGCGATGAG
AGGGAGGTGCCTGTGGGAGCCGTGCTGGTGCTGAACAATAGAGTGATCGG
CGAGGGCTGGAACAGAGCCATCGGCCTGCACGACCCAACAGCCCATGCC
GAAATTATGGCCCTGAGACAGGGCGGCCTGGTCATGCAGAACTACAGACT
GATTGACGCCACCCTGTACGTGACATTCGAGCCTTGCGTGATGTGCGCCG
GCGCCATGATCCACTCTAGGATCGGCCGCGTGGTGTTTGGCGTGAGGAAC
GCAAAAACCGGCGCCGCAGGCTCCCTGATGGACGTGCTGCACTACCCCGG
CATGAATCACCGCGTCGAAATTACCGAGGGAATCCTGGCAGATGAATGTG
CCGCCCTGCTGTGCTATTTCTTTCGGATGCCTAGACAGGTGTTCAATGCTC
AGAAGAAGGCCCAGAGCTCCACCGACTCCGGAGGATCTAGCGGAGGCTC
CTCTGGCTCTGAGACACCTGGCACAAGCGAGAGCGCAACACCTGAAAGCT
CCGGAGGATCTAGCGGAGGCTCCTCTGGTGGTTCTTCTGGTGGTTCTAGCG
GGGGCAGCAGCGGGGGGTCAATGGGTCCCGACATCGTGATGACCCAGAG
CCCCAGCAGCCTGAGCGCCAGCGTGGGCGACCGCGTGACCATCACCTGCC
GCAGCAGCACCGGCGCCGTGACCACCAGCAACTACGCCAGCTGGGTGCA
GGAGAAGCCCGGCAAGCTGTTCAAGGGCCTGATCGGCGGCACCAACAAC
CGCGCCCCCGGCGTGCCCAGCCGCTTCAGCGGCAGCCTGATCGGCGACAA
GGCCACCCTGACCATCAGCAGCCTGCAGCCCGAGGACTTCGCCACCTACT
TCTGCGCCCTGTGGTACAGCAACCACTGGGTGTTCGGCCAGGGCACCAAG
GTGGAGCTGAAGCGCGGCGGCGGCGGCAGCGGCGGCGGCGGCAGCGGCG
GCGGCGGCAGCAGCGGCGGCGGCAGCGAGGTGAAGCTGCTGGAGAGCGG
CGGCGGCCTGGTGCAGCCCGGCGGCAGCCTGAAGCTGAGCTGCGCCGTGA
GCGGCTTCAGCCTGACCGACTACGGCGTGAACTGGGTGCGCCAGGCCCCC
GGCCGCGGCCTGGAGTGGATCGGCGTGATCTGGGGCGACGGCATCACCGA
CTACAACAGCGCCCTGAAGGACCGCTTCATCATCAGCAAGGACAACGGCA
AGAACACCGTGTACCTGCAGATGAGCAAGGTGCGCAGCGACGACACCGC
CCTGTACTACTGCGTGACCGGCCTGTTCGACTACTGGGGCCAGGGCACCC
TGGTGACCGTGAGCAGCTACCCATACGATGTTCCAGATTACGCTGGTTCTG

GCGGCTCAAAAAGAACCGCCGACGGCAGCGAATTCGAGCCCAAGAAGAA
GAGGAAAGTCTAA

pVax-PAH-WT
ATGTCCACTGCGGTCCTGGAAAACCCAGGCTTGGGCAGGAAACTCTCTGA
CTTTGGACAGGAAACAAGCTATATTGAAGACAACTGCAATCAAAATGGTG
CCATATCACTGATCTTCTCACTCAAAGAAGAAGTTGGTGCATTGGCCAAA
GTATTGCGCTTATTTGAGGAGAATGATGTAAACCTGACCCACATTGAATCT
AGACCTTCTCGTTTAAAGAAAGATGAGTATGAATTTTTCACCCATTTGGAT
AAACGTAGCCTGCCTGCTCTGACAAACATCATCAAGATCTTGAGGCATGA
CATTGGTGCCACTGTCCATGAGCTTTCACGAGATAAGAAGAAAGACACAG
TGCCCTGGTTCCCAAGAACCATTCAAGAGCTGGACAGATTTGCCAATCAG
ATTCTCAGCTATGGAGCGGAACTGGATGCTGACCACCCTGGTTTTAAAGA
TCCTGTGTACCGTGCAAGACGGAAGCAGTTTGCTGACATTGCCTACAACT
ACCGCCATGGGCAGCCCATCCCTCGAGTGGAATACATGGAGGAAGAAAA
GAAAACATGGGGCACAGTGTTCAAGACTCTGAAGTCCTTGTATAAAACCC
ATGCTTGCTATGAGTACAATCACATTTTTCCACTTCTTGAAAAGTACTGTG
GCTTCCATGAAGATAACATTCCCCAGCTGGAAGACGTTTCTCAATTCCTGC
AGACTTGCACTGGTTTCCGCCTCCGACCTGTGGCTGGCCTGCTTTCCTCTC
GGGATTTCTTGGGTGGCCTGGCCTTCCGAGTCTTC**C**ACTGCACACAGTACA
TCAGACATGGATCCAAGCCCATGTATACCCCCGAACCTGACATCTGCCAT
GAGCTGTTGGGACATGTGCCCTTGTTTTCAGATCGCAGCTTTGCCCAGTTT
TCCCAGGAAATTGGCCTTGCCTCTCTGGGTGCACCTGATGAATACATTGAA
AAGCTCGCCACAATTTACTGGTTTACTGTGGAGTTTGGGCTCTGCAAACAA
GGAGACTCCATAAAGGCATATGGTGCTGGGCTCCTGTCATCCTTTGGTGA
ATTACAGTACTGCTTATCAGAGAAGCCAAAGCTTCTCCCCCTGGAGCTGG
AGAAGACAGCCATCCAAAATTACACTGTCACGGAGTTCCAGCCCCTGTAT
TACGTGGCAGAGAGTTTTAATGATGCCAAGGAGAAAGTAAGGAACTTTGC
TGCCACAATACCTCGGCCCTTCTCAGTTCGCTACGACCCATACACCCAAAG
GATTGAGGTCTTGGACAATACCCAGCAGCTTAAGATTTTGGCTGATTCCAT
TAACAGTGAAATTGGAATCCTTTGCAGTGCCCTCCAGAAAATAAAGTAA

pVax-PAH-H264Y
ATGTCCACTGCGGTCCTGGAAAACCCAGGCTTGGGCAGGAAACTCTCTGA
CTTTGGACAGGAAACAAGCTATATTGAAGACAACTGCAATCAAAATGGTG
CCATATCACTGATCTTCTCACTCAAAGAAGAAGTTGGTGCATTGGCCAAA
GTATTGCGCTTATTTGAGGAGAATGATGTAAACCTGACCCACATTGAATCT
AGACCTTCTCGTTTAAAGAAAGATGAGTATGAATTTTTCACCCATTTGGAT
AAACGTAGCCTGCCTGCTCTGACAAACATCATCAAGATCTTGAGGCATGA
CATTGGTGCCACTGTCCATGAGCTTTCACGAGATAAGAAGAAAGACACAG
TGCCCTGGTTCCCAAGAACCATTCAAGAGCTGGACAGATTTGCCAATCAG
ATTCTCAGCTATGGAGCGGAACTGGATGCTGACCACCCTGGTTTTAAAGA
TCCTGTGTACCGTGCAAGACGGAAGCAGTTTGCTGACATTGCCTACAACT
ACCGCCATGGGCAGCCCATCCCTCGAGTGGAATACATGGAGGAAGAAAA

GAAAACATGGGGCACAGTGTTCAAGACTCTGAAGTCCTTGTATAAAACCC
ATGCTTGCTATGAGTACAATCACATTTTTCCACTTCTTGAAAAGTACTGTG
GCTTCCATGAAGATAACATTCCCCAGCTGGAAGACGTTTCTCAATTCCTGC
AGACTTGCACTGGTTTCCGCCTCCGACCTGTGGCTGGCCTGCTTTCCTCTC
GGGATTTCTTGGGTGGCCTGGCCTTCCGAGTCTTC**T**ACTGCACACAGTACA
TCAGACATGGATCCAAGCCCATGTATACCCCCGAACCTGACATCTGCCAT
GAGCTGTTGGGACATGTGCCCTTGTTTTCAGATCGCAGCTTTGCCCAGTTT
TCCCAGGAAATTGGCCTTGCCTCTCTGGGTGCACCTGATGAATACATTGAA
AAGCTCGCCACAATTTACTGGTTTACTGTGGAGTTTGGGCTCTGCAAACAA
GGAGACTCCATAAAGGCATATGGTGCTGGGCTCCTGTCATCCTTTGGTGA
ATTACAGTACTGCTTATCAGAGAAGCCAAAGCTTCTCCCCCTGGAGCTGG
AGAAGACAGCCATCCAAAATTACACTGTCACGGAGTTCCAGCCCCTGTAT
TACGTGGCAGAGAGTTTTAATGATGCCAAGGAGAAAGTAAGGAACTTTGC
TGCCACAATACCTCGGCCCTTCTCAGTTCGCTACGACCCATACACCCAAAG
GATTGAGGTCTTGGACAATACCCAGCAGCTTAAGATTTTGGCTGATTCCAT
TAACAGTGAAATTGGAATCCTTTGCAGTGCCCTCCAGAAAATAAAGTAA
